# Carbonic anhydrases reduce the acidity of the tumor microenvironment, promote immune infiltration, decelerate tumor growth, and improve survival in ErbB2/HER2-enriched breast cancer

**DOI:** 10.1186/s13058-023-01644-1

**Published:** 2023-04-25

**Authors:** Soojung Lee, Nicolai J. Toft, Trine V. Axelsen, Maria Sofia Espejo, Tina M. Pedersen, Marco Mele, Helene L. Pedersen, Eva Balling, Tonje Johansen, Mark Burton, Mads Thomassen, Pernille Vahl, Peer Christiansen, Ebbe Boedtkjer

**Affiliations:** 1 Department of Biomedicine, Aarhus University, Hoegh-Guldbergs Gade 10, Building 1115, DK-8000 Aarhus C, Denmark; 2 Department of Surgery, Randers Regional Hospital, Randers, Denmark; 3 Department of Pathology, Randers Regional Hospital, Randers, Denmark; 4 Department of Clinical Genetics, University of Southern Denmark, Odense, Denmark; 5 Clinical Genome Center, University and Region of Southern Denmark, Odense, Denmark; 6 Department of Clinical Medicine, University of Southern Denmark, Odense, Denmark; 7 Department of Pathology, Aarhus University Hospital, Aarhus, Denmark; 8 Department of Plastic and Breast Surgery, Aarhus University Hospital, Aarhus, Denmark

**Keywords:** Acetazolamide, Acidosis, Breast cancer, Carbonic anhydrases, ErbB2, HER2, Immuno-oncology, Metabolism, Perfusion, Tumor microenvironment

## Abstract

**Background:**

Carbonic anhydrases catalyze CO_2_/HCO_3_^–^ buffer reactions with implications for effective H^+^ mobility, pH dynamics, and cellular acid–base sensing. Yet, the integrated consequences of carbonic anhydrases for cancer and stromal cell functions, their interactions, and patient prognosis are not yet clear.

**Methods:**

We combine (a) bioinformatic analyses of human proteomic data and bulk and single-cell transcriptomic data coupled to clinicopathologic and prognostic information; (b) ex vivo experimental studies of gene expression in breast tissue based on quantitative reverse transcription and polymerase chain reactions, intracellular and extracellular pH recordings based on fluorescence confocal microscopy, and immunohistochemical protein identification in human and murine breast cancer biopsies; and (c) in vivo tumor size measurements, pH-sensitive microelectrode recordings, and microdialysis-based metabolite analyses in mice with experimentally induced breast carcinomas.

**Results:**

Carbonic anhydrases—particularly the extracellular isoforms *CA4*, *CA6*, *CA9*, *CA12*, and *CA14*—undergo potent expression changes during human and murine breast carcinogenesis. In patients with basal-like/triple-negative breast cancer, elevated expression of the extracellular carbonic anhydrases negatively predicts survival, whereas, surprisingly, the extracellular carbonic anhydrases positively predict patient survival in HER2/ErbB2-enriched breast cancer. Carbonic anhydrase inhibition attenuates cellular net acid extrusion and extracellular H^+^ elimination from diffusion-restricted to peripheral and well-perfused regions of human and murine breast cancer tissue. Supplied in vivo, the carbonic anhydrase inhibitor acetazolamide acidifies the microenvironment of ErbB2-induced murine breast carcinomas, limits tumor immune infiltration (CD3^+^ T cells, CD19^+^ B cells, F4/80^+^ macrophages), lowers inflammatory cytokine (*Il1a*, *Il1b*, *Il6*) and transcription factor (*Nfkb1*) expression, and accelerates tumor growth. Supporting the immunomodulatory influences of carbonic anhydrases, patient survival benefits associated with high extracellular carbonic anhydrase expression in HER2-enriched breast carcinomas depend on the tumor inflammatory profile. Acetazolamide lowers lactate levels in breast tissue and blood without influencing breast tumor perfusion, suggesting that carbonic anhydrase inhibition lowers fermentative glycolysis.

**Conclusions:**

We conclude that carbonic anhydrases (a) elevate pH in breast carcinomas by accelerating net H^+^ elimination from cancer cells and across the interstitial space and (b) raise immune infiltration and inflammation in ErbB2/HER2-driven breast carcinomas, restricting tumor growth and improving patient survival.

**Supplementary Information:**

The online version contains supplementary material available at 10.1186/s13058-023-01644-1.

## Introduction

**Fig. 1:**
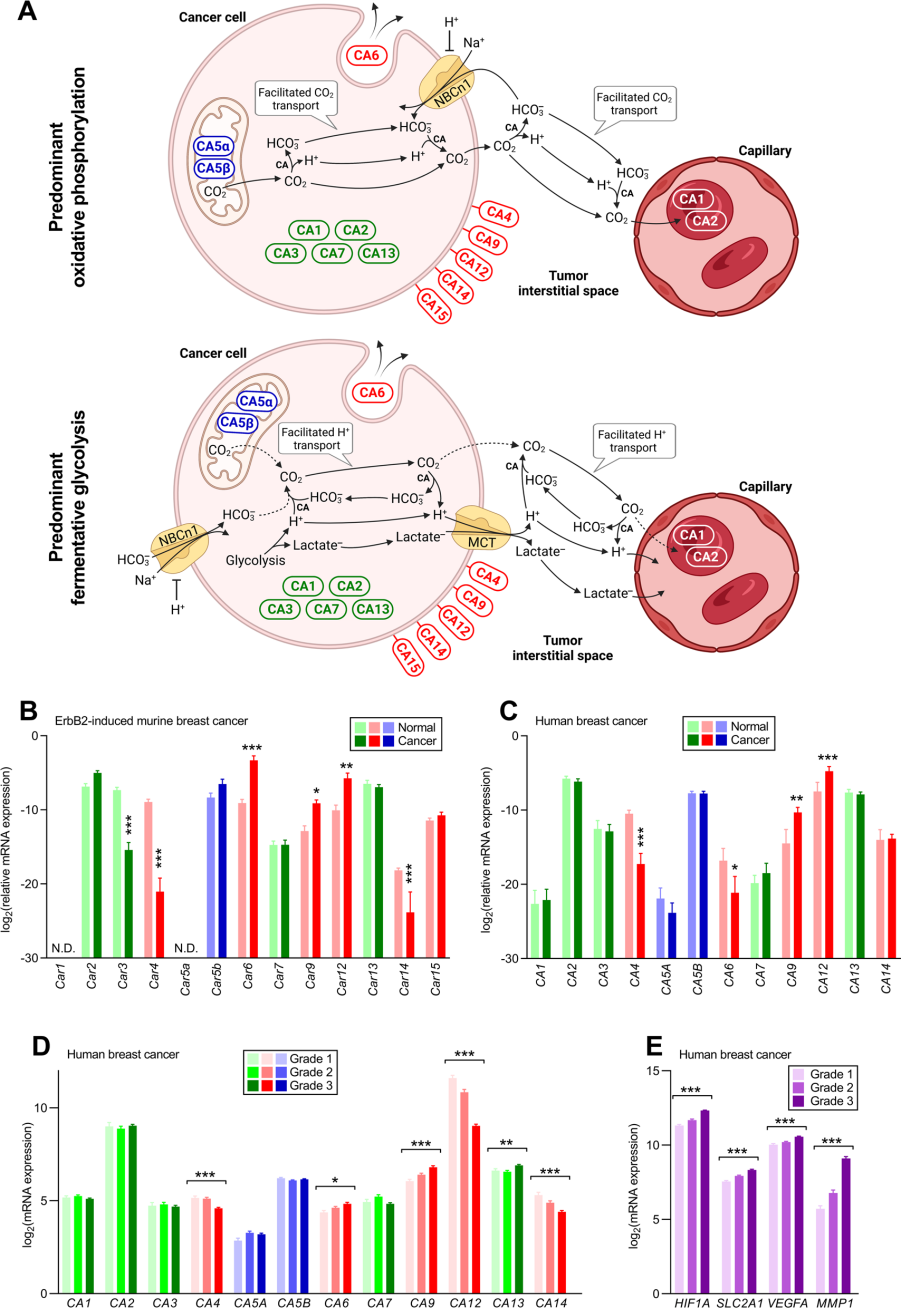
Carbonic anhydrases—particularly, extracellularly localized isoforms—undergo dynamic expression changes during human and murine breast carcinogenesis. **A** Schematic of how carbonic anhydrases based on their reported expression patterns can accelerate elimination of acidic waste products from solid cancer tissue. Separate cartoons show conditions dominated by oxidative phosphorylation (upper panel) and fermentative glycolysis (lower panel). Cytosol: CA1, CA2, CA3, CA7, and CA13. Mitochondria: CA5α and CA5β. Membrane-associated extracellular-facing: CA4, CA9, CA12, CA14, and CA15. Secreted: CA6. Reactions labeled CA are catalyzed by carbonic anhydrases. Note also the allosteric inhibition of NBCn1 exerted by extracellular H^+^ [23]. MCT, monocarboxylate transporter. The schematic was created with BioRender.com. **B + C** Transcript levels for carbonic anhydrases in murine ErbB2-induced (**B**, n = 7–8) and human (**C**, n = 9–14) breast cancer. Expression levels are reported relative to the reference genes *ACTB*/*Actb* and *RPS18*/*Rps18*. Data were compared by mixed model analyses followed by Šidák’s post-tests. *P < 0.05, **P < 0.01, ***P < 0.001 *vs.* normal. N.D., not detectable. **D + E** Expression of carbonic anhydrases (**D**) and known hypoxia-responsive gene products (**E**) in human breast cancer tissue (n = 82–446) of increasing malignancy grade. The data cover the whole breadth of malignant breast tumors without stratification for molecular subtype. Data from the GPL570 platform were extracted from the GENT2 database [38] and compared by one-way ANOVA for trend followed by Holm–Bonferroni adjustment for multiple comparisons. *P < 0.05, **P < 0.01, ***P < 0.001 *vs.* slope = 0; see Additional file 1: Table S4 for details. We did not evaluate expression of the isoforms CA8, CA10, and CA11 that do no not show carbonic anhydrase catalytic activity

The acidity of the tumor microenvironment fundamentally impacts cancer and stromal cell functions, their interactions, and the selection pressure and adaptive processes that shape malignant progression [1, 2]. Cancer cell metabolism liberates H^+^ when CO_2_ derived from oxidative phosphorylation undergoes hydration (Fig. 1A, upper panel) and when glucose converts to lactate through fermentative glycolysis (Fig. 1A, lower panel). The magnitude of the local acid load and the mechanisms of acid–base regulation and sensing vary between tumors of different origins, molecular subtypes, and malignancies [3–6]. The acid–base composition of the tumor microenvironment holds considerable promise for prognostic prediction and development of new therapeutic approaches, yet we still lack mechanistic insight to rationally design targeted interventions.

Intracellular acid from cancer cells is first exported via transport proteins in the plasma membrane to the tumor interstitial space and then transferred to nearby blood vessels (Fig. 1A). Acid–base heterogeneity within tumors can develop, for instance, due to spatiotemporal variation in metabolic activity, expression of acid–base transporters, diffusion hindrances, and perfusion. Net acid extrusion from breast cancer cells occurs mostly via the Na^+^, HCO_3_^–^-cotransporter NBCn1/SLC4A7 (Fig. 1A) and Na^+^/H^+^-exchanger NHE1/SLC9A1 [7–9]. During fermentative glycolysis (Fig. 1A, lower panel), monocarboxylate transporters (MCTs) also contribute with coupled cellular efflux of H^+^ and lactate [6]. Interpatient heterogeneity in cytosolic pH, the capacity for Na^+^,HCO_3_^–^-cotransport and Na^+^/H^+^-exchange, and the expression of NBCn1 and NHE1 in breast tumors independently predict proliferative activity in primary carcinomas, the occurrence of regional lymph node metastasis, and patient survival [3].

Carbonic anhydrases—that catalyze the reaction CO_2_ + H_2_O $\rightleftarrows$ HCO_3_^–^ + H^+^—are interesting in oncology because they aid the multi-step process of transferring acid generated from metabolic activity within solid cancer tissue to the bloodstream (Fig. 1A). Furthermore, well-tolerated small molecule carbonic anhydrase inhibitors are in current clinical use for diverse indications, including glaucoma, idiopathic intracranial hypertension, seizures, congestive heart failure, and mountain sickness [10–12]. Targeting individual carbonic anhydrase isoforms with distinct cellular expression patterns and localization to the cytosol (CA1, CA2, CA3, CA7, CA13), mitochondrial matrix (CA5α, CA5β), or extracellular space (membrane tethered: CA4, CA9, CA12, CA14 or secreted: CA6) offers attractive prospects for modifying acid–base conditions in select cell types or subcellular compartments [13]. The membrane-tethered carbonic anhydrases with catalytic domains directed to the interstitial space have potential to minimize spatiotemporal pH heterogeneity within solid tumors by accelerating the CO_2_/HCO_3_^–^ buffer reaction and increasing the effective H^+^ mobility [14]. Previous studies suggest that CA9 also has pro-carcinogenic effects that are at least partly independent of its carbonic anhydrase activity, involve cell adhesion function, and can be influenced by shedding of the extracellular domain [15–17].

Whereas CA9 and CA12 are prognostic markers and proposed pharmacological targets in breast cancer [18, 19], the consequences of these and other carbonic anhydrases in specific breast cancer molecular subtypes remain unclear [6, 20]. It is particularly unclear how carbonic anhydrases influence interactions between cancer cells and stromal cells—including the vasculature and immune system—in tumors differing in metabolic and proliferative activity, immunogenicity, and underlying oncogenic pathways. Acid–base-dependent changes in the tumor vasculature and immune cell function are prominent ex vivo [21, 22], but their consequences within the tumor microenvironment demand further investigation.

The pronounced diffusion limitations within solid cancer tissue—in combination with focal accumulation of carbonic anhydrase activity and pH-mediated allosteric regulation of acid–base transporters [23, 24]—result in compartmentalized pH dynamics that fundamentally influence tumor biology. Therefore, to resolve the functional contribution of carbonic anhydrases during carcinogenesis and cancer progression, it is crucial to study cancer tissue in vivo or under ex vivo conditions that maintain a realistic 3-dimensional structure and relevant stromal component. Here, we achieve this goal by (a) investigating carbonic anhydrase expression and function in organoids freshly isolated from human and murine breast cancer tissue and matched normal breast tissue, (b) in vivo studies in a murine breast cancer model, and (c) evaluating human bulk and single-cell transcriptomic data coupled to clinical and pathological information.

Breast cancer is a highly heterogeneous disease. Stratification based on receptor expression profiles (e.g., HER2/ErbB2, estrogen, progesterone) identifies molecular subtypes with overlapping characteristics in underlying oncogenic mechanisms and malignant behavior. Thus, this categorization provides an important tool for unmasking disease mechanisms and elucidating therapeutic consequences that may differ between subsets of breast cancer patients.

We demonstrate that carbonic anhydrases facilitate elimination of acidic metabolites from breast cancer tissue and that their inhibition intensifies the acidity of the tumor microenvironment. In murine ErbB2-induced and human HER2-enriched breast carcinomas, extracellular carbonic anhydrases increase immune cell infiltration and cytokine expression, decelerate tumor growth, and improve survival.

## Materials and methods

### Tissue biopsies

We obtained viable human tissue biopsies from 23 patients undergoing breast-conserving lumpectomy at the Department of Surgery, Randers Regional Hospital or the Department of Breast and Plastic Surgery, Aarhus University Hospital, Denmark [9, 25, 26]. Additional file 1: Table S1 summarizes the clinical and pathological patient characteristics.

We acquired murine breast tissue from a mouse model with breast epithelial overexpression of ErbB2 [8, 26, 27]. These mice develop primary breast tumors at an average age of approximately 6 months [8].

### Organoid isolation

Biopsies of breast cancer tissue and matched normal breast tissue were finely chopped in phosphate-buffered saline (PBS) before transfer to advanced DMEM/F12 culture medium (Life Technologies, Denmark) supplemented with 10% fetal bovine serum (Biochrom, Germany), 1% Glutamax (Gibco, Invitrogen, Denmark), and 450 IU/mL collagenase type 3 (Worthington Biochemicals, USA). The tissue was incubated in this solution for 4 h (mouse) or overnight (human) on a shaking table at 60 rpm in a 37 °C atmosphere of 5% CO_2_/balance air. At the end of tissue digestion, organoids around 150 µm in diameter were sedimented by gravitational forces for 20 min [7, 25]. We immediately investigated the freshly isolated organoids experimentally in order to avoid culture-induced phenotypical changes.

### Quantitative reverse transcription and polymerase chain reaction

Freshly isolated organoids from human and murine breast tissue were stored at –80 °C until they were disrupted in RLT lysis buffer using Qiagen TissueLyser (Denmark). Total RNA was purified using RNeasy Mini Kit—either manually or in an automated QIAcube system (Qiagen, Denmark)—DNase-treated or cleaned with a gDNA eliminator column, and reverse transcribed using Reverse Transcriptase III (Invitrogen, CA, USA) or SuperScript Retrotranscriptase IV (ThermoFisher), RNase inhibitor Superase (Invitrogen), and random decamer primers (Eurofins Genomics, Germany) in a VWR peqSTAR thermocycler. We determined RNA and DNA concentrations with a Picodrop spectrophotometer (ThermoFisher Scientific) and controlled for genomic amplification by including experiments without reverse transcriptase added.

The quantitative polymerase chain reactions evaluating carbonic anhydrase expression were performed in duplicate with Maxima Hot Start Taq DNA Polymerase and a Stratagene MX3000P system (AH Diagnostics, Denmark) using Brilliant II SYBR Green QPCR Master Mix (Stratagene, 600828-51). Primer sequences are reported in Additional file 1: Tables S2 and S3. The reactions consisted of 1 cycle at 95 °C for 10 min, followed by 50 cycles at 95 °C for 30 s, 55 °C (for mouse) or 56 °C (for human) for 30 s, and 72 °C for 30 s, and finally 1 cycle at 95 °C for 1 min, 55 °C for 30 s, and 95 °C for 30 s.

We evaluated cytokine and transcription factor expression based on predesigned TaqMan primers and probes purchased from ThermoFisher Scientific (*Tnf*: Mm00443258_m1, *Nfkb1*: Mm00476361_m1, *Il1a*: Mm00439620_m1, *Il1b*: Mm00434228_m1, *Il6*: Mm00446190_m1, *Tgfb1*: Mm01178820_m1, *Rps18*: Mm02601777_g1, *Actb*: Mm02619580_g1) using DreamTaq Polymerase (ThermoFisher). The reactions run in the Stratagene MX3000P system consisted of 1 cycle at 95 °C for 10 min followed by 60 cycles at 95 °C for 30 s, 55 °C 60 s, and 72 °C for 60 s.

We assessed relative mRNA levels from the 2^−ΔCT^ value where ΔC_T_ is the cycle threshold difference between the gene of interest and the mean of the reference genes (*ACTB*/*Actb* and *RPS18*/*Rps18*).

### In vitro pH recordings using tissue lysates

Freshly isolated organoids from mouse breast cancer tissue were disrupted in 0.1% Triton X-100 using pellet pestles, suspended in 20 mM HEPES solution, and added 5 µM of the pH-sensitive dual-excitation fluorophore 2′,7′-bis-(2-carboxyethyl)-5-(and-6)-carboxyfluorescein (BCECF) acid (B1151; Invitrogen). We monitored pH using a Photon Technology International spectrophotometer (USA) that collected emission light at 535 nm during alternating excitation at 490 and 440 nm. We transferred 1.5 mL cell suspension to a cuvette and analyzed the rate of acidification in response to addition of 0.5 mL distilled water saturated with 100% CO_2_ [14] under control conditions and in the presence of the carbonic anhydrase inhibitors acetazolamide and 4-(aminomethyl)benzenesulfonamide (AMB).

### Intracellular and extracellular pH measurements in organoids

We performed pH measurements in thin optical slices based on confocal fluorescence microscopy of freshly isolated organoids from human and murine breast cancer tissue.

To measure intracellular pH (pH_i_), we loaded freshly isolated organoids with 20 µM of the acetoxymethyl form of the pH-sensitive dual-emission fluorophore carboxy-SNARF-1 (C-1272; Invitrogen) for 20 min at 37 °C. After washout of the fluorophore, the organoids were excited at 488 nm and emission light was collected simultaneously in the wavelength range between 505–600 nm and at wavelengths longer than 615 nm.

To measure extracellular pH (pH_o_), the freshly isolated organoids were loaded with 10 µM of the fluorescein-based dual-excitation fluorophore Fluorescein DHPE (Invitrogen, #F362) for 20 min at 37 °C. Following fluorophore washout, the organoids were excited alternatingly at 458 and 488 nm, with emission light collected at 530 nm.

Both pH_i_ and pH_o_ recordings were performed using an Axiovert 200 M Zeiss confocal microscope with an LSM Pascal exciter. We converted the carboxy-SNARF-1 and Fluorescein DHPE fluorescence emission ratios to pH using the high-[K^+^] nigericin calibration technique [28]. Net acid extrusion activity after NH_4_^+^-prepulse-induced intracellular acidification [29] was calculated as pH_i_ recovery rate × buffering capacity. Based on the assumption that NH_3_ is in equilibrium across cell membranes, we estimated intracellular intrinsic buffering capacities from the change in pH_i_ upon addition and subsequent washout of NH_4_Cl in absence of CO_2_/HCO_3_^–^ [30]. We calculated intracellular buffering capacity contributed by CO_2_/HCO_3_^–^ using the equation $\beta_{{{\text{CO}}_{2} /{\text{HCO}}_{3}^{ - } }} = 2.3 \times \left[ {{\text{HCO}}_{3}^{ - } } \right]_{{\text{i}}}$ [30, 31]. We determined the Na^+^-dependent net acid extrusion activity during NH_4_^+^-prepulse-induced intracellular acidification based on the difference in pH_i_ recovery rate with and without bath Na^+^.

CO_2_/HCO_3_^−^‐containing salt solutions used during recordings of pH_i_ and pH_o_ were composed of (in mM [32]): 138 Na^+^, 4 K^+^, 1.6 Ca^2+^, 1.2 Mg^2+^, 142 Cl^−^, 22 HCO_3_^−^, 1.2 SO_4_^2−^, 1.18 H_2_PO_4_^−^, 2 HEPES, 5.5 glucose, and 0.03 EDTA. HCO_3_^−^-containing solutions were bubbled with 5% CO_2_/balance air. Cl^–^ replaced HCO_3_^–^ in HCO_3_^–^-free solutions that were bubbled with nominally CO_2_-free air. Probenecid (5 µM) was added to all solutions to inhibit fluorophore extrusion by the organic anion transporter. We adjusted pH of the final solutions to 7.4 at 37 °C. We used 100 µM acetazolamide for combined inhibition of intracellular and extracellular carbonic anhydrases, 30 µM AMB for selective inhibition of extracellular carbonic anhydrases [33, 34], and 200 µM FC5-207A (generously provided by Dr. Claudiu Supuran, University of Florence, Italy) for selective inhibition of CA9 [35].

### Measurements of metabolites and tumor perfusion in vivo

Mice under pentobarbital anesthesia (induction: 2 mg, maintenance: ~ 0.5 mg every 30 min) were placed on a heating pad, endotracheally intubated, and ventilated under capnographic control (HSE-HA Capnography Type 340; Harvard apparatus) of the expiratory end-tidal CO_2_-fraction at 3.5–3.8% [36]. Drugs or equivalent volumes of vehicle (0.9% NaCl or 25% DMSO in PBS) were administered through intraperitoneal injections. The anesthetized mice were evaluated by one of the following procedures: (a) Mice were instrumented with microdialysis probes (CMA 20 Elite, 4 mm membrane length; CMA Microdialysis AB, Sweden) positioned in tumor and normal breast tissue. An initial dose of 50 mg/kg acetazolamide or vehicle was followed after 30 min by a second dose of 25 mg/kg acetazolamide or vehicle. The microdialysis probes were continuously perfused at 0.5 µL/min, and starting one hour after the initial acetazolamide injection, 6 µL microdialysate was collected to allow for analysis of [glucose] and [lactate] using an ISCUSflex microdialysis analyzer (M Dialysis, Sweden). Calculation of interstitial concentrations relied on relative analyte recoveries determined in vitro for each microdialysis probe. (b) We studied tumor perfusion using Laser Doppler Flowmetry (moorVMS-LDF; Moor Instruments, UK), which provides a combined measure of the concentration and velocity of red blood cells [37]. Recordings were performed from the tumor and a control region in the lateral groin corresponding to the arterial supply for the lower limb. Mice were injected with 50 mg/kg acetazolamide, 5 mg/kg of the α_1_-adrenoceptor agonist phenylephrine, or corresponding vehicle.

### Measurements of tumor pH in vivo

We evaluated the pH consequences of acute carbonic anhydrase inhibition in vivo by first injecting tumor-bearing mice with 50 mg/kg acetazolamide or vehicle intraperitoneally. Then, after 5 min, the mice received an injection of ketamine (80 mg/kg Ketaminol® vet) and xylazine (8 mg/kg Narcoxyl® vet) to induce anesthesia. The mice were placed on a heating pad; and after a total of 30 min, we exposed the tumor through a small incision and used a glass microelectrode (pH 500; Unisense, Denmark), as previously described [36], to record pH during progressive 1-mm steps into the tumor. The reference electrode was placed in the intraperitoneal space. We report pH at the peritoneal surface, at 4 mm depth into the tumor, and at the most acidic tumor location encountered (denoted tumor “core”).

### Measurement of tumor growth in vivo

To ensure early detection of tumor development, we initiated twice-weekly palpations when the mice with breast epithelial overexpression of ErbB2 reached an age of 4 months. From the time of tumor detection, the mice were treated with one daily intraperitoneal injection of 40 mg/kg acetazolamide or vehicle (25% DMSO in PBS). We measured tumor width (W) and length (L) with electronic calipers twice a week throughout the treatment period and calculated the corresponding tumor volume (V) as V = π/6 × W^2^ × L. We subtracted 2 mm from each tumor dimension measured in vivo in order to control for skin thickness. Acetazolamide treatment continued for up to 4 weeks; but to comply with animal ethics guidelines, experiments were terminated and the mice euthanized if the estimated tumor volume approached 900 µL.

### Immunohistochemistry

At the end of the in vivo treatment period—consisting of up to 4 weeks of daily acetazolamide injections (40 mg/kg/day) or equivalent volume of vehicle—the breast cancer tissue was immersion fixed for 60 min in 4% neutral-buffered formaldehyde (VWR, Denmark). After paraffin embedding, the tissue was cut to 3 µm thick sections. The deparaffinized and rehydrated slides were treated with 3% H_2_O_2_ for 10–20 min to inhibit endogenous peroxidase activity. For epitope retrieval, the slides were microwave heated at 600 W for 1 × 10 min (CD105, F4/80), 2 × 10 min (Ki67) or 1 × 20 min (CD3, CD19) in citrate buffer at pH 6.0. The slides were blocked with 10% goat serum (CD3, CD19, F4/80) or 1% (Ki67) or 5% (CD105) bovine serum albumin in PBS (Ki67, CD3, F4/80, CD19) or Tris-buffered saline (TBS) containing 2% Tween 20 (CD105) for 20 min (Ki67) or 1 h (F4/80, CD105, CD3, CD19) at room temperature. The slides were then incubated overnight at 4℃ in PBS containing 1% (CD3, CD19, F4/80, Ki67) or 5% (CD105) bovine serum albumin, 2% Tween 20 (CD105) or 0.1% Triton X-100 (Ki67, CD3, CD19, F4/80), and one of the following primary antibodies: rabbit anti-Ki67 (Abcam #ab16667, diluted 1:200), goat anti-CD105 (R&D Systems #AF1320, diluted 1:50), rabbit anti-F4/80 (Cell Signaling Technology #70,076, diluted 1:250), rabbit anti-CD3 (Cell Signaling Technology #99,940, diluted 1:150), or rabbit anti-CD19 (Cell Signaling Technology #90,176, diluted 1:800). After thorough washing for 3 × 5 min in PBS with (CD3, CD19) or without (Ki67, F4/80) 0.1% Tween 20 or in TBS containing 2% Tween 20 (CD105), slides were incubated with species-matched horseradish peroxidase-conjugated secondary antibodies (Cell Signaling Technology; anti-rabbit #7074S or anti-goat #63,707, diluted 1:1000) for 30–60 min. After renewed washing in PBS (Ki67, CD3, CD19, F4/80) or Tween 20-containing TBS (CD105), bound antibody was detected with 3,3′-diaminobenzidine (DAB; Sigma-Aldrich #D3939), and slides were counterstained with hematoxylin. We produced full-slide scans using an Olympus VS120 virtual slide microscope and counted positive and negative cells and vascular structures within the epithelial mass using QuPath 0.3.2 software (University of Edinburgh, UK). Similar threshold and detection criteria were applied to all slides stained with the same primary antibody.

### Transcript levels and survival data from human breast cancer

We first retrieved information on transcript levels through the online GENT2 database [38]. We separately analyzed data from the Affymetrix Human Genome U133 (GPL96) and U133 Plus 2.0 (GPL570) GeneChip arrays. Data extracted from the GPL96 platform provided information covering 4,293 breast cancer samples and 92 samples of normal breast tissue from studies of the Gene Expression Omnibus series: GSE1456, GSE1561, GSE2361, GSE2603, GSE3494, GSE3726, GSE4611, GSE4922, GSE5327, GSE5364, GSE5462, GSE5847, GSE6532, GSE6772, GSE6883, GSE7390, GSE9574, GSE9662, GSE11121, GSE11965, GSE12093, GSE12237, GSE12630, GSE15852, GSE16873, GSE2034, GSE22093, GSE23988, GSE24185, GSE24509, GSE25066, GSE31519, GSE32072, GSE36774, GSE45255, GSE46184, GSE48984, GSE68892, GSE83232, and GSE92697. Data extracted from the GPL570 platform provided information covering 5,574 breast cancer samples (including 725 samples characterized for malignancy grade) and 475 samples of normal breast tissue from the studies E-TAMB-276, GSE2109, GSE3744, GSE5460, GSE5764, GSE6532, GSE7307, GSE7515, GSE7904, GSE8977, GSE9195, GSE10281, GSE10780, GSE10810, GSE11001, GSE12276, GSE12763, GSE13671, GSE13787, GSE16391, GSE16446, GSE17907, GSE18331, GSE18728, GSE18864, GSE19615, GSE19697, GSE20086, GSE20685, GSE20713, GSE21422, GSE21653, GSE22035, GSE22513, GSE22544, GSE23177, GSE23720, GSE25407, GSE26457, GSE26639, GSE26910, GSE27120, GSE29832, GSE31138, GSE31192, GSE31448, GSE32646, GSE35603, GSE36245, GSE36774, GSE42568, GSE43358, GSE43346, GSE43365, GSE43502, GSE45827, GSE46222, GSE47109, GSE47389, GSE48391, GSE51238, GSE51452, GSE52322, GSE54002, GSE58812, GSE61304, GSE65216, GSE66162, GSE70233, GSE71258, GSE73613, GSE75333, and GSE76275.

Using the PAM50 Breast Cancer Intrinsic Classifier [39], we next performed expression and survival analyses within individual breast cancer molecular subtypes [40, 41]. In addition to seven microarray datasets from studies by van de Vijver et al. [42], Guo et al. [43], Calza et al. [44], GSE1992, GSE2034, GSE11121, and GSE3143—that we previously used to evaluate gene expression by a similar approach [3, 5]—we retrieved 31 additional datasets through the online Kaplan–Meier Plotter at kmplot.com [45]: E-MTAB-365, GSE12093, GSE12276, GSE1456, GSE16391, GSE16446, GSE17705, GSE17907, GSE19615, GSE20685, GSE20711, GSE21653, GSE25066, GSE2603, GSE26971, GSE2990, GSE31519, GSE3494, GSE37946, GSE42568, GSE45255, GSE4611, GSE46184, GSE48390, GSE5327, GSE61304, GSE65194, GSE6532, GSE69031, GSE7390, GSE9195. For studies measuring gene expression with multiple probes per gene, we collapsed multiple expression values using the maximum mean probe intensity. We then separately standardized each dataset across samples and combined the datasets into one matrix that we subjected to a second round of cross-sample standardization. Based on this standardized expression matrix covering 5,889 patients, we compared carbonic anhydrase expression levels between breast cancer molecular subtypes and conducted survival analyses. We calculated z-score = (individual expression score–population mean)/SD and constructed Kaplan–Meier survival curves for each carbonic anhydrase isoform by dividing individuals between groups with high (z-score > 0) and low (z-score < 0) mRNA level. For each breast cancer molecular subtype, we also separately calculated the average mRNA expression level for the carbonic anhydrase isoforms that localize to the extracellular space (*CA4*, *CA6*, *CA9*, *CA12*, *CA14*), cytosol (*CA1*, *CA2*, *CA3*, *CA7*, *CA13*), and mitochondrial matrix (*CA5A*, *CA5B*) and produced Kaplan–Meier survival curves based on three groups showing high (z-score > 0.25), moderate (0.25 > z-score > –0.25), and low (z-score < –0.25) carbonic anhydrase expression. In general, patients were censored on the date of the last follow-up visit, upon death from causes other than breast cancer, recurrence of local or regional disease, or development of a second primary cancer, including contralateral breast cancer [42].

### Single-cell RNA sequencing data

We explored levels of carbonic anhydrase transcripts in individual cell types from human breast cancer tissue based on the online Single Cell Portal, which is hosted by the Broad Institute and accessible at https://singlecell.broadinstitute.org. We retrieved data from a recent single-cell RNA sequencing study that offers a comprehensive transcriptional atlas of the cellular architecture of human breast cancer [46].

### Proteomic data

We extracted proteomic data from three breast cancer studies (PDC000120, PDC000173, and PDC000408) via the online Proteomic Data Commons portal of the National Cancer Institute, National Institutes of Health, USA, which is accessible at https://pdc.cancer.gov. We used log_2_ ratios based on unshared peptides only.

### Statistics

Data are given as mean ± SEM unless otherwise specified. The n-values stated in the figure legends represent biological replicates, i.e., they specify the number of patients or animals investigated. The investigators were blinded during in vivo treatments, measurements, and analyses. We compared one parameter between two groups by two-tailed Student’s *t* tests and between more than two groups by one-way ANOVA followed by linear trend analysis (ordered groups) or Šidák’s post-tests (unordered groups). We corrected for multiple comparisons based on the Holm–Bonferroni method. We evaluated effects of two or three independent variables on a dependent variable using two- and three-way ANOVA or in case of missing values by mixed model statistics, followed by Šidák’s post-tests. We compared two Kaplan–Meier curves by Mantel–Cox and Gehan–Breslow–Wilcoxon tests and three ordered Kaplan–Meier curves by log-rank tests for trend. Right-skewed data were log- or square root-transformed before comparisons. Statistical analyses were performed with GraphPad Prism 9.4.1.

## Results

We perform experimental studies in vivo and in vitro on mice and human tissue biopsies and evaluate them in light of clinically annotated transcript information to explore the role of carbonic anhydrases in breast cancer.

### Expression of carbonic anhydrase isoforms during breast carcinogenesis and in breast carcinomas of increasing malignancy grade

Human and murine breast cancer tissue and corresponding normal breast tissue express transcripts for multiple carbonic anhydrase isoforms (Fig. 1B-D) with known localization in the cytosol (*CA1*, *CA2*/*Car2*, *CA3*/*Car3*, *CA7*/*Car7*, *CA13*/*Car13*), mitochondria (*CA5A*, *CA5B*/*Car5b*), and extracellular space either membrane-tethered (*CA4*/*Car4*, *CA9*/*Car9*, *CA12*/*Car12*, *CA14*/*Car14*, *Car15*) or secreted (*CA6*/*Car6*).

From quantitative RT-PCR experiments, we compare expression levels in breast cancer tissue with that in matched normal breast tissue (Fig. 1B,C). We observe the most pronounced expression changes during breast carcinogenesis within the group of membrane-tethered extracellular carbonic anhydrases. Most markedly *CA9*/*Car9* and *CA12*/*Car12* are upregulated, whereas *CA4*/*Car4* is downregulated in human as well as murine breast cancer tissue (Fig. 1B,C). In addition, we observe downregulation of *Car14* specifically in the murine breast cancer tissue (Fig. 1B). Interestingly, the secreted *CA6*/*Car6* is downregulated in the human breast cancer tissue (Fig. 1C) but upregulated in the murine breast cancer tissue (Fig. 1B). Among the intracellular carbonic anhydrases, only the cytosolic *Car3* shows significant expression changes during breast carcinogenesis, as it is downregulated in murine breast cancer tissue compared to normal breast tissue (Fig. 1B).

We next evaluate whether carbonic anhydrase expression varies as function of breast cancer malignancy grade (Fig. 1D). To obtain a sufficiently large sample size for these analyses, we extract and compare publically available data on transcript levels. Whereas the cytosolic and mitochondrial carbonic anhydrases show no (*CA1*, *CA2*, *CA3*, *CA5A*, *CA5B*, *CA7*) or only minimal (*CA13*) regulation between breast cancers of different malignancy grades, the secreted (*CA6*) and extracellular-facing, membrane-tethered (*CA4*, *CA9*, *CA12*, *CA14*) carbonic anhydrases are markedly altered in expression between high and low malignancy grade breast cancer (Fig. 1D and Additional file 1: Table S4). Apart from *CA9*—which is known to be HIF1α-responsive and follows the increasing expression pattern of other known hypoxia-regulated genes from low to high malignancy grade breast cancer (Fig. 1E and Additional file 1: Table S4)—the other extracellular-facing, membrane-tethered carbonic anhydrases (*CA4*, *CA12*, *CA14*) show a consistent pattern of marked downregulation from low to high grade breast cancers (Fig. 1D and Additional file 1: Table S4). In addition to the well-known influence of hypoxia on HIF1α at the level of protein stability, transcriptional regulation of *HIF1A* plays a considerable role in cancer [47, 48]. Indeed, we see that *HIF1A* mRNA levels follow the same expression pattern as the tested HIF1α-responsive genes (Fig. 1E).

### Extracellular and cytosolic carbonic anhydrases are predominantly in cancer epithelial and endothelial cells

**Fig. 2:**
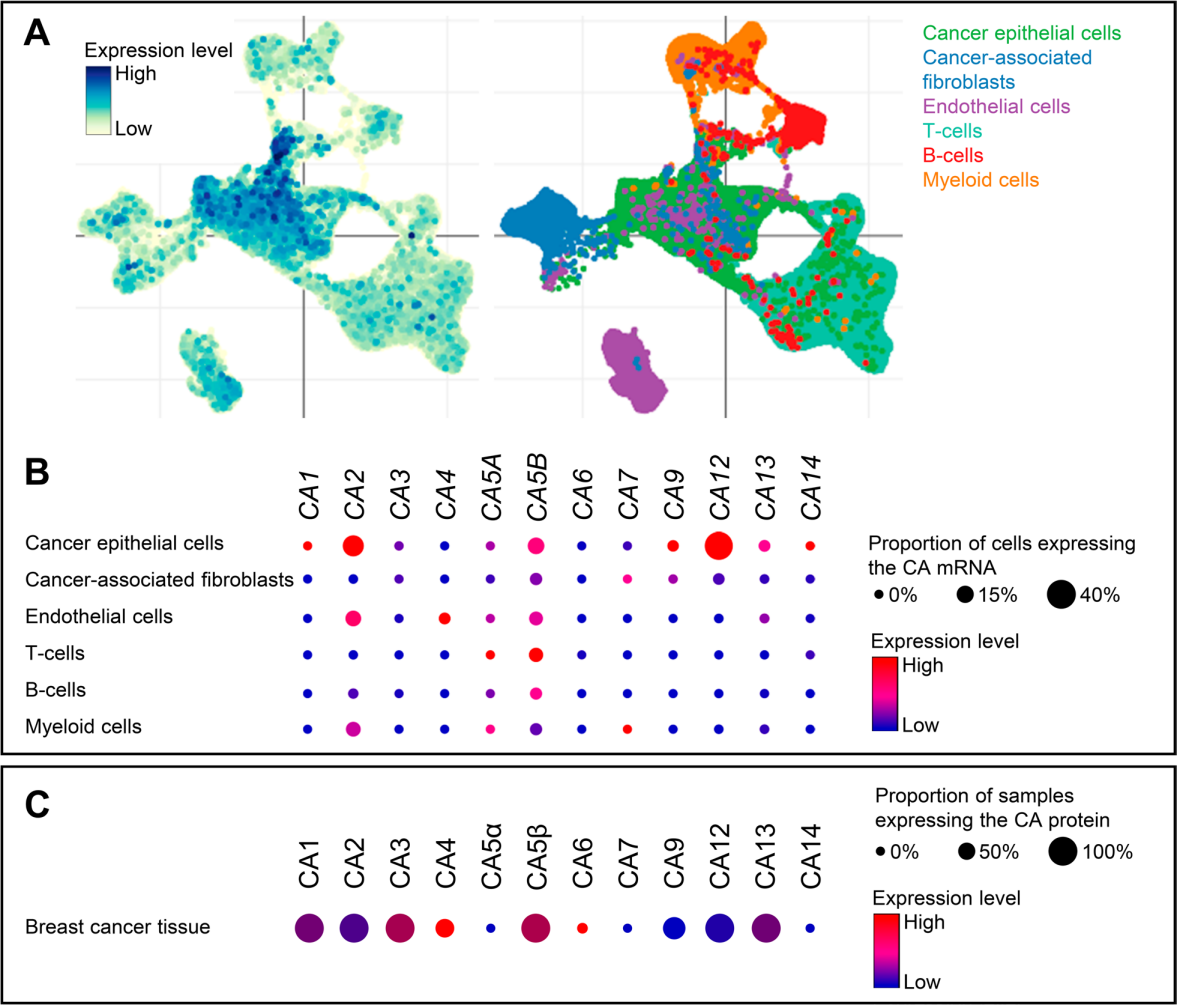
In human breast cancer tissue, mitochondrial *CA5A* and *CA5B* are ubiquitously expressed across cell types, whereas the non-mitochondrial carbonic anhydrase isoforms predominate in cancer epithelial and endothelial cells, except for *CA7* expressed solely by cancer-associated fibroblasts and myeloid cells. **A** Overall carbonic anhydrase expression intensities illustrated as t-SNE plot (left) with corresponding clustered cell types (right). **B** Dot plot showing the cell type-specific pattern of carbonic anhydrase expression in human breast cancer. The reported single-cell sequencing data cover 24,489 cancer epithelial cells, 6573 cancer-associated fibroblasts, 7605 endothelial cells, 35,214 T cells, 3206 B cells, and 9675 myeloid cells. Data were extracted from the online Single Cell Portal hosted by the Broad Institute [46]. **C** Dot plot showing the protein expression levels of carbonic anhydrases across 294 breast cancer samples. Data were extracted from the online Proteomic Data Commons portal hosted by the National Cancer Institute. CA, carbonic anhydrase

Breast cancer tissue consists of multiple cell types organized in a complex 3-dimensional arrangement. To determine the cellular distribution patterns of the individual carbonic anhydrases, we explore single-cell RNA sequencing data from human breast cancer tissue (Fig. 2).

As shown in Fig. 2B, the mitochondrial *CA5A* and *CA5B* show relatively similar expression levels across cancer epithelial cells, endothelial cells, cancer-associated fibroblasts, and immune cells (T cells, B cells, and myeloid cells). Most of the other carbonic anhydrase isoforms—especially *CA1*, *CA2*, *CA3*, *CA9*, *CA12*, *CA13*, and *CA14*—are expressed at highest level in breast cancer epithelial cells (Fig. 2B). Notable exceptions to this pattern are *CA4*, which is detected only in breast cancer endothelial cells, and *CA7*, which is expressed exclusively in cancer-associated fibroblasts and myeloid cells (Fig. 2B). Other relevant expression signals include *CA2* and *CA13* in endothelial cells and *CA2* in myeloid cells (Fig. 2B).

Based on proteomic data from human biopsies, we additionally confirm the expression of cytosolic (CA1, CA2, CA3, CA13), mitochondrial (CA5β), and extracellular (CA4, CA6, CA9, CA12) carbonic anhydrases in breast cancer tissue (Fig. 2C).

### The patterns of carbonic anhydrase expression relate to patient survival

**Fig. 3:**
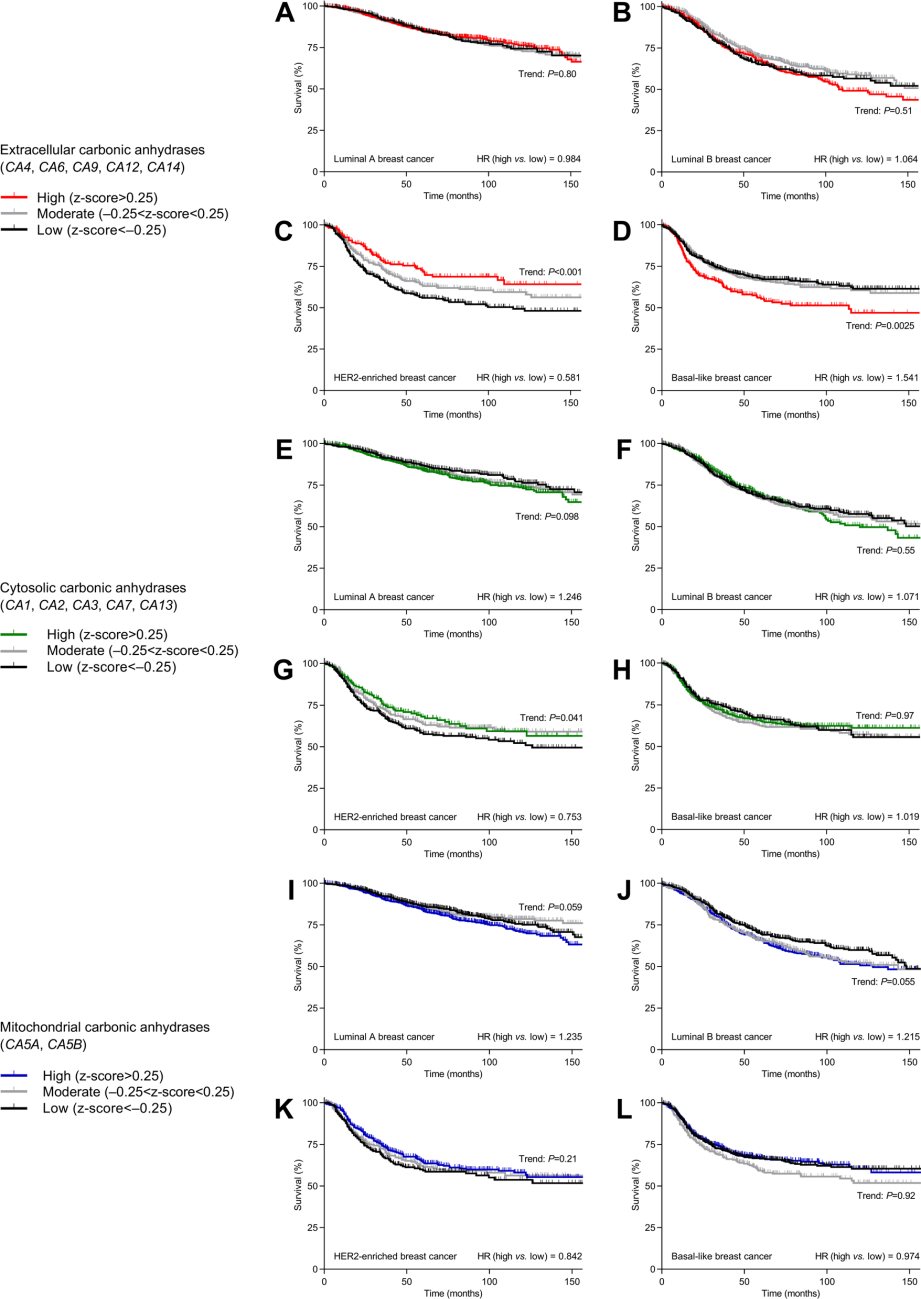
Extracellular and cytosolic carbonic anhydrases positively predict prognosis in HER2-enriched breast cancer, whereas extracellular carbonic anhydrases negatively predict prognosis in Basal-like breast cancer. **A–L** Survival curves for breast cancer patients (n = 860–2053) stratified for average mRNA expression of extracellular (**A**–**D**), cytosolic (**E**–**H**), and mitochondrial (**I**–**L**) carbonic anhydrases within each breast cancer molecular subtype. The ticks on the curves indicate censored subjects. Data were compared by log-rank test for trend. HR, hazard ratio

**Fig. 4:**
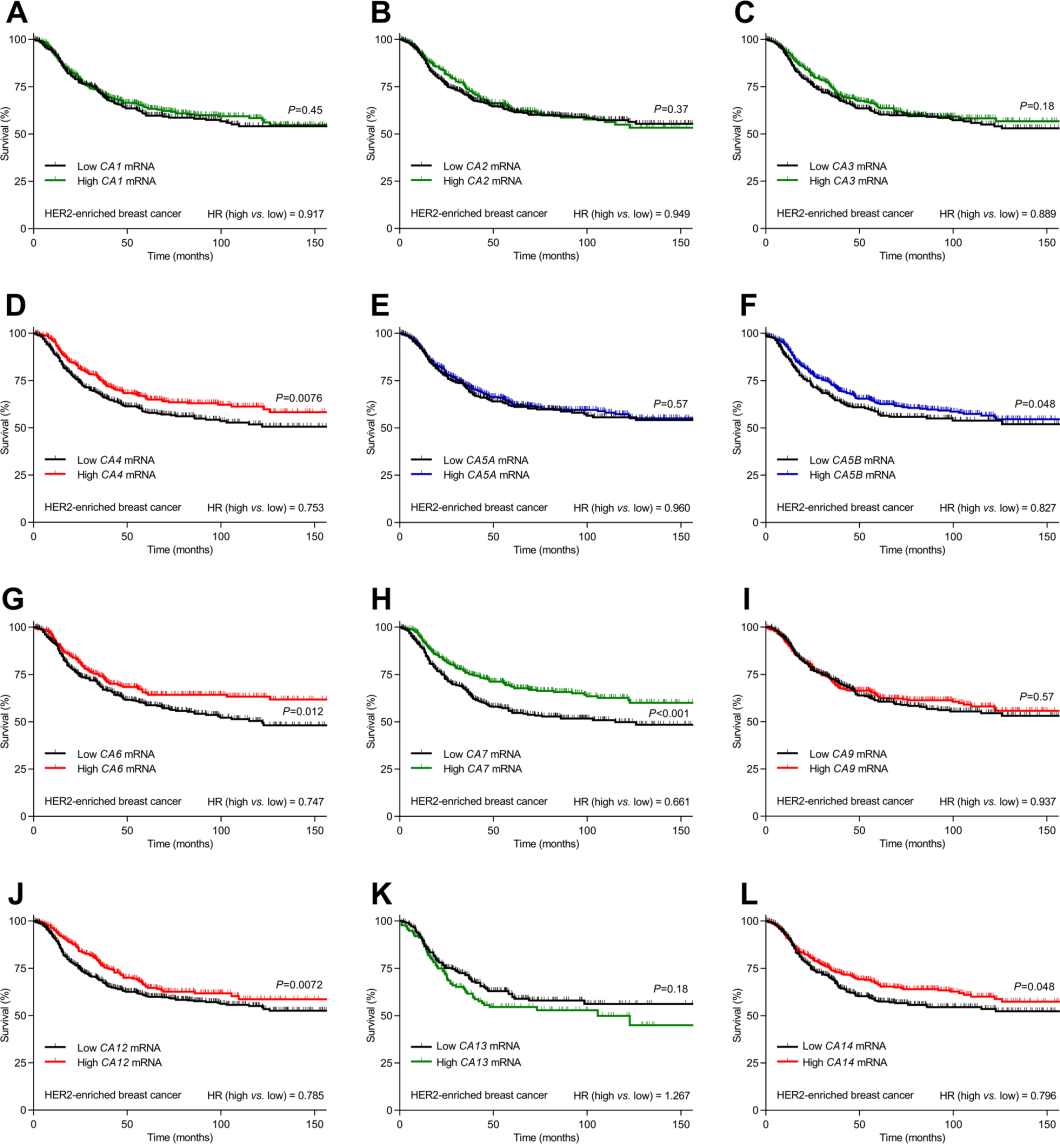
Carbonic anhydrase isoforms CA4, CA6, CA12, and CA14 with known extracellular expression, CA5β with known mitochondrial expression, and CA7 with known cytosolic expression positively predict prognosis for patients with HER2-enriched breast cancer. **A–L** Survival curves for patients (n = 358–860) with HER2-enriched breast cancer stratified for mRNA expression of each carbonic anhydrase isoform. The color coding of the curves distinguishes carbonic anhydrase isoforms with known extracellular or secreted (red), cytosolic (green), and mitochondrial (blue) expression. The ticks on the curves indicate censored subjects. Low and high mRNA levels refer to z-scores below and above zero, respectively. Data were compared by Mantel–Cox and Gehan–Breslow–Wilcoxon tests. HR, hazard ratio

**Fig. 5:**
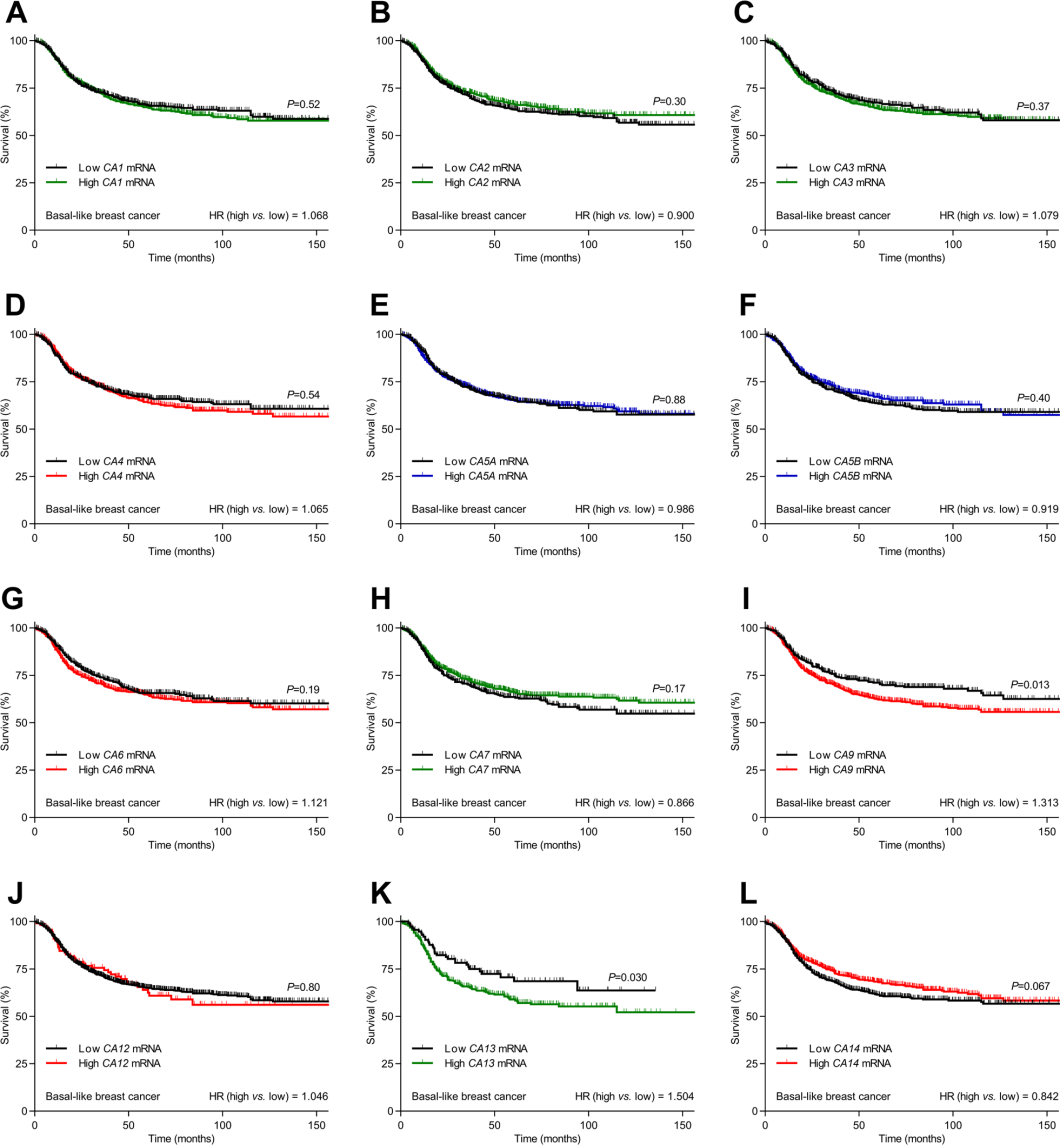
Carbonic anhydrase isoforms CA9, with known extracellular expression, and CA13, with known cytosolic expression, negatively predict prognosis for patients with Basal-like breast cancer. **A-L** Survival curves for patients (n = 442–1188) with Basal-like breast cancer stratified for mRNA expression of each carbonic anhydrase isoform. The color coding of the curves distinguishes carbonic anhydrase isoforms with known extracellular or secreted (red), cytosolic (green), and mitochondrial (blue) expression. The ticks on the curves indicate censored subjects. Low and high mRNA levels refer to z-scores below and above zero, respectively. Data were compared by Mantel–Cox and Gehan–Breslow–Wilcoxon tests. HR, hazard ratio

The influence of acid–base conditions differs between breast cancer molecular subtypes relying on distinct oncogenic mechanisms [3, 5] and displaying distinct expression patterns for individual carbonic anhydrase isoforms (Additional file 1: Fig. S1). Therefore, we evaluate patient survival separately for Luminal A, Luminal B, HER2-enriched, and Basal-like breast cancer. We first study how survival of breast cancer patients relates to the expression of carbonic anhydrases with distinct subcellular localization (extracellular, cytosolic, mitochondrial; Fig. 3) and perform similar analyses for each of the carbonic anhydrase isoforms individually (Figs. 4 and 5 and Additional file 1: Figs. S2 and S3).

The extracellular carbonic anhydrases are associated with dramatic survival effects in breast cancer patients but show surprising heterogeneity among the breast cancer molecular subtypes (Fig. 3A-D). Whereas high expression levels of extracellular carbonic anhydrases predict improved survival in HER2-enriched breast cancer (hazard ratio (HR) = 0.581, Fig. 3C), they associate with reduced survival in Basal-like breast cancer (HR = 1.541, Fig. 3D) and show no overall effect on survival in Luminal A (HR = 0.984, Fig. 3A) and Luminal B (HR = 1.064, Fig. 3B) breast cancer. The individual extracellular carbonic anhydrase isoforms display rather uniform survival effects in HER2-enriched breast cancer (all with HR < 1; Fig. 4D, G, I, J, and L). In contrast, *CA9* drives most of the negative survival effect of extracellular carbonic anhydrases in Basal-like breast cancer (HR = 1.313; Fig. 5I).

We observe overall survival effects of cytosolic carbonic anhydrases (Fig. 3E-H) only in HER2-enriched breast cancer where high expression levels associate with improved survival (HR = 0.753, Fig. 3G). This effect of the cytosolic carbonic anhydrases in HER2-enriched breast cancer is driven almost entirely by *CA7* (HR = 0.661, Fig. 4H), whereas the cytosolic *CA13* shows an equally substantial negative predictive effect in Basal-like breast cancer (HR = 1.502, Fig. 5K). The distinct effect of *CA7* compared to other cytosolic carbonic anhydrase isoforms in HER2-enriched breast cancer is consistent with the expression of *CA7* exclusively in cancer-associated fibroblasts and myeloid cells, whereas expression of the other cytosolic carbonic anhydrase isoforms (*CA1*, *CA2*, *CA3*, and *CA13*) is most prominent in cancer epithelial and endothelial cells (Fig. 2B). In Luminal A breast cancer, the cytosolic carbonic anhydrases *CA1* (HR = 1.295, Additional file 1: Fig. S2A), *CA2* (HR = 1.340, Additional file 1: Fig. S2B), and *CA3* (HR = 0.753, Additional file 1: Fig. S2C) associate significantly with survival, and a similar influence of *CA2* is observed in Luminal B breast cancer (HR = 1.205, Additional file 1: Fig. S3B).

We observe no significant overall survival effects of the mitochondrial carbonic anhydrases (Fig. 3I-L), although there is a strong tendency toward reduced survival in Luminal A (HR = 1.235, *P* = 0.059, Fig. 3I) and Luminal B (HR = 1.215, *P* = 0.055, Fig. 3J) breast cancer. Yet, *CA5B* is associated with improved survival in HER2-enriched breast cancer (HR = 0.827, Fig. 4F).

The positive prognostic influence of carbonic anhydrases in HER2-enriched breast cancer (Figs. 3 and 4) is unexpected. Consistent with our findings from Basal-like breast cancer, previous literature [18] emphasizes, in particular, that CA9 negatively predicts survival (Figs. 3 and 5). To explore further the consequences of carbonic anhydrases in breast cancer and focus explicitly on our unexplained findings from HER2-enriched breast cancer, we next perform functional experiments on human breast cancer tissue and in a mouse model of ErbB2-induced breast cancer.

### Acetazolamide and AMB inhibit carbonic anhydrase activity in breast cancer tissue lysates

**Fig. 6:**
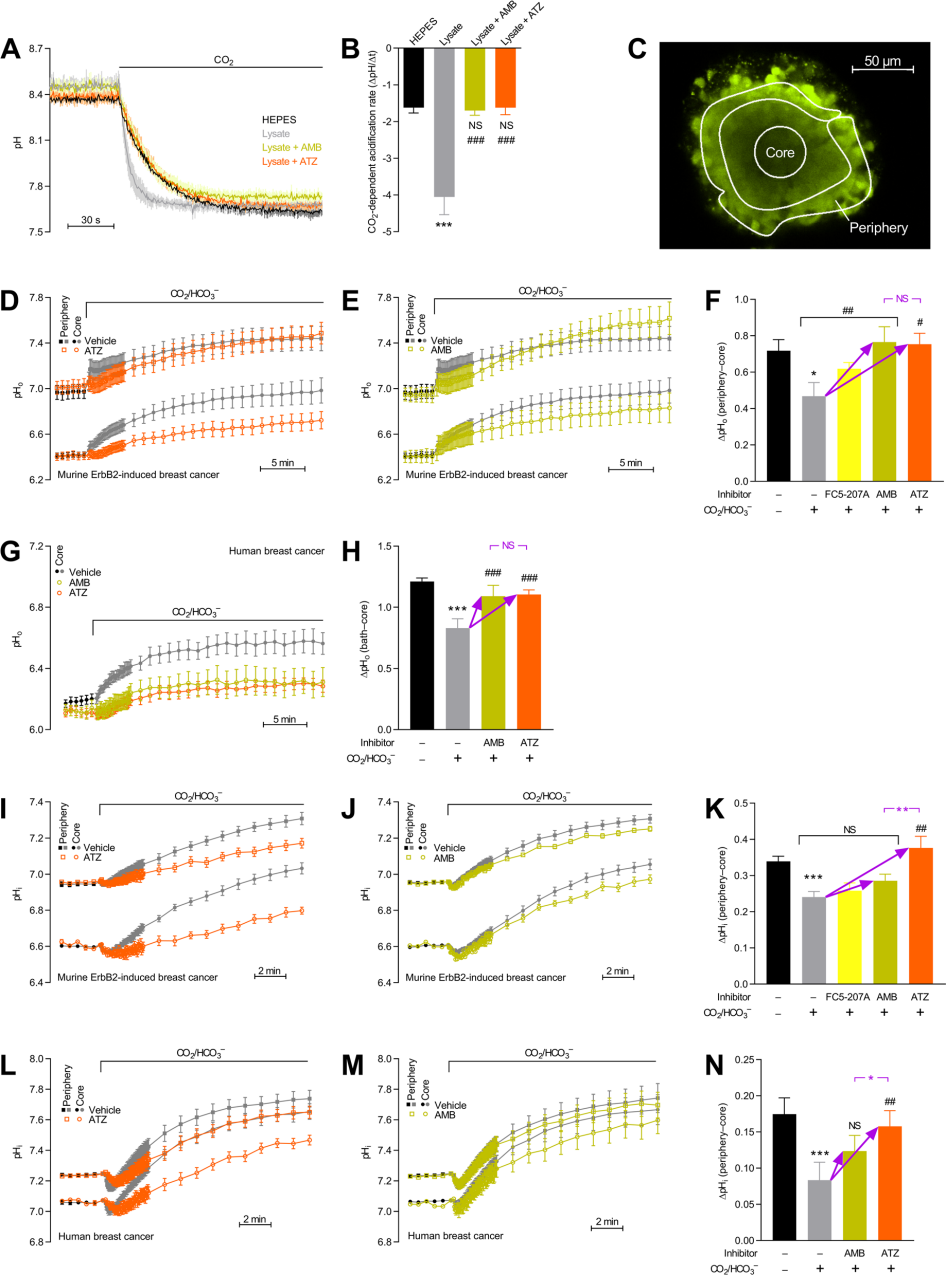
Carbonic anhydrases fundamentally modify pH dynamics in breast cancer tissue, inhibiting net acid extrusion from cancer cells and lowering interstitial net H^+^ elimination from the core to the periphery. **A + B** Average pH traces (**A**) and quantified rates of acidification (**B**) in response to application of CO_2_ to a HEPES-buffered CO_2_/HCO_3_^–^-free solution. We show how lysates of ErbB2-induced murine breast cancer tissue and the carbonic anhydrase inhibitors acetazolamide (ATZ, 100 µM) and 4-(aminoethyl)benzenesulfonamide (AMB, 30 µM) influence the rate of CO_2_ hydration (n = 4). Data were compared by one-way ANOVA followed by Šidák’s post-test. ***P < 0.001, NS: not significantly different *vs.* HEPES. ^###^*P* < 0.001 *vs.* lysate. **C** Confocal image acquired at the equatorial plane of an organoid loaded with the pH-sensitive fluorophore carboxy-SNARF-1. The white delineations illustrate exemplary regions of interest used to quantify pH dynamics in the organoid core and periphery. **D-H** Extracellular (surface) pH dynamics in response to acute exposure of murine ErbB2-induced (**D**–**F**, n = 8–14) and human (**G** + **H**, n = 8–9) breast cancer organoids to CO_2_/HCO_3_^–^ relative to the initial mean value in absence of CO_2_/HCO_3_^–^. Panel **F** shows quantified pH_o_ differences between the core and periphery of organoids and how they are modified by CO_2_/HCO_3_^–^ and carbonic anhydrase inhibition; panel **H** shows similar information regarding the pH_o_ differences between the organoid core and experimental bath. FC5-207A was used at a concentration of 200 µM. *P < 0.05, ***P < 0.001 *vs.* CO_2_/HCO_3_^–^-free. ^#^*P* < 0.05, ^###^*P* < 0.001 *vs.* CO_2_/HCO_3_^–^. ^##^*P* < 0.01 *vs.* slope = 0 by linear trend analysis. Purple “NS” (= not significantly different) report the results of statistical tests comparing the effect of ATZ to that of AMB in the presence of CO_2_/HCO_3_^–^ as indicated by the purple arrows. **I-N** Intracellular pH dynamics in response to acute exposure of murine ErbB2-induced (**I**–**K**, n = 6–26) and human (**L**-**N**, n = 8) breast cancer organoids to CO_2_/HCO_3_^–^ relative to the initial mean value in absence of CO_2_/HCO_3_^–^. Panel **K** and **N** show pH_i_ differences between the core and periphery of organoids and how they are modified by CO_2_/HCO_3_^–^ and carbonic anhydrase inhibition. ***P < 0.001 *vs.* CO_2_/HCO_3_^–^-free. ^##^*P* < 0.01 *vs.* CO_2_/HCO_3_^–^. NS: not significantly different *vs.* slope = 0 by linear trend analysis (panel K) or *vs.* CO_2_/HCO_3_^–^ (panel N). Purple stars (*P < 0.05, **P < 0.01) report the results of statistical tests comparing the effect of ATZ to that of AMB in the presence of CO_2_/HCO_3_^–^ as indicated by the purple arrows. Data in panel F, H, K, and N were compared by one-way ANOVA followed by Šidák’s post-test or linear trend analysis. The ATZ- and AMB-induced pH-changes were compared by unpaired two-tailed Student’s *t* tests

We first test effects of pharmacological carbonic anhydrase inhibitors on pH regulation in breast cancer tissue. We use the membrane-permeable acetazolamide for combined inhibition of intra- and extracellular carbonic anhydrases and the membrane-impermeable AMB for selective inhibition of extracellular carbonic anhydrases. At the applied concentrations, acetazolamide (100 µM) and AMB (30 µM) are both non-selective with respect to the carbonic anhydrase isoforms expressed in breast tumors; as confirmed by our observation that acetazolamide and AMB both fully inhibit the accelerating effect of tumor lysates on CO_2_-induced acidification (Fig. 6A,B).

### The CO_2_/HCO_3_^–^ buffer alkalinizes the extracellular tumor microenvironment of breast carcinomas and this effect requires extracellular carbonic anhydrase activity

Based on pH-sensitive fluorophores loaded into organoids freshly isolated from human and murine breast cancer tissue (Fig. 6C), we next evaluate whether carbonic anhydrase activity is important for control of pH_i_ and pH_o_.

We first use Fluorescein DHPE that reports pH at the outer cell surface and immerse murine breast cancer organoids in CO_2_/HCO_3_^−^‐free solution (Fig. 6D, E). Under these conditions, we observe a large pH_o_ gradient from the acidic core to a more alkaline periphery (Fig. 6F). When we next switch to a CO_2_/HCO_3_^−^‐containing solution, both core and periphery alkalinize but the effect is greater at the core than at the periphery (Fig. 6D, E). The resulting decrease in pH_o_ gradient from the core to the periphery (Fig. 6F) illustrates that CO_2_/HCO_3_^–^ is an important mobile buffer pair [49] that accelerates transfer of interstitial acid from the organoid core through the periphery to the bath. We speculate that this accelerated transfer of acid by facilitated H^+^ diffusion on the CO_2_/HCO_3_^–^ buffer requires extracellular carbonic anhydrase activity (Fig. 1A). Indeed, we observe that acetazolamide or AMB added to the bath solution abolishes the decrease in core-to-periphery pH_o_ gradient when murine ErbB2-induced breast cancer organoids are exposed to CO_2_/HCO_3_^–^-containing solution (Fig. 6F). We also test the CA9-specific inhibitor FC5-207A. In the presence of 200 µM FC5-207A, the pH_o_ gradient from the organoid core to periphery (Fig. 6F) is intermediate to that under control CO_2_/HCO_3_^–^ conditions and in the presence of AMB. These findings are consistent with FC5-207A acting on only one out of several extracellular carbonic anhydrases (Figs. 1 and 2).

Due to poor penetration of Fluorescein DHPE into the human breast cancer organoids, we are unable to record simultaneously pH_o_ at the core and periphery. However, when we optimize recordings at the organoid core (Fig. 6G) and compare the measurements to the bath pH of 7.4, we see effects of CO_2_/HCO_3_^–^ and carbonic anhydrase inhibitors in the human organoids (Fig. 6H) identical to those observed in murine organoids (Fig. 6F).

### The CO_2_/HCO_3_^–^ buffer causes intracellular alkalinization in breast carcinomas and this effect requires intracellular carbonic anhydrase activity

Interstitial acidification predictably leads to intracellular acidification [7, 8]. It is therefore expected that the graded extracellular acidification from the organoid periphery toward the core is paralleled by a similar gradient in pH_i_. Indeed, as illustrated in Fig. 6I-N, cancer cells at the core of both human and murine breast cancer organoids are much more acidic than at the periphery, especially when no CO_2_/HCO_3_^–^ is present in the bath medium. After we switch to a solution containing CO_2_/HCO_3_^–^, pH_i_ rises both at the core and periphery (Fig. 6I, J, L, and M). This elevation of pH_i_ can be explained by the dual role of CO_2_/HCO_3_^–^, providing substrate for net acid extrusion via Na^+^,HCO_3_^–^-cotransport and enhancing the effective H^+^ mobility in the cytosol and interstitial space (Fig. 1A). Because the CO_2_/HCO_3_^–^-induced intracellular alkalinization is greater at the core than at the periphery of the organoids, the pH_i_ difference between the core and periphery decreases upon addition of CO_2_/HCO_3_^–^ (Fig. 6K, N). This differential effect of CO_2_/HCO_3_^–^ at the core and periphery of breast cancer organoids is abrogated when intra- and extracellular carbonic anhydrases are inhibited with acetazolamide but not when only extracellular carbonic anhydrases or CA9 are inhibited with AMB and FC5-207A, respectively (Fig. 6I-N).

### Intracellular carbonic anhydrase activity accelerates cellular net acid extrusion in breast cancer tissue

At least two different mechanisms have been proposed whereby carbonic anhydrase activity can facilitate transport of acids and bases across cell membranes. Firstly, extracellular carbonic anhydrases can attenuate the interstitial acidification in breast cancer tissue (Fig. 6D-H) and thereby relieve inhibition of membrane acid–base transporters. Specifically, the Na^+^/H^+^-exchanger NHE1 and Na^+^,HCO_3_^–^-cotransporter NBCn1 crucial for net acid extrusion from breast cancer tissue [7–9, 50] are inhibited in response to extracellular acidification [23, 51, 52]. Secondly, intracellular carbonic anhydrase activity has been found to maximize net acid extrusion [53, 54] by facilitating net H^+^ delivery to the internal membrane surface of H^+^ and HCO_3_^–^ transporters.

**Fig. 7:**
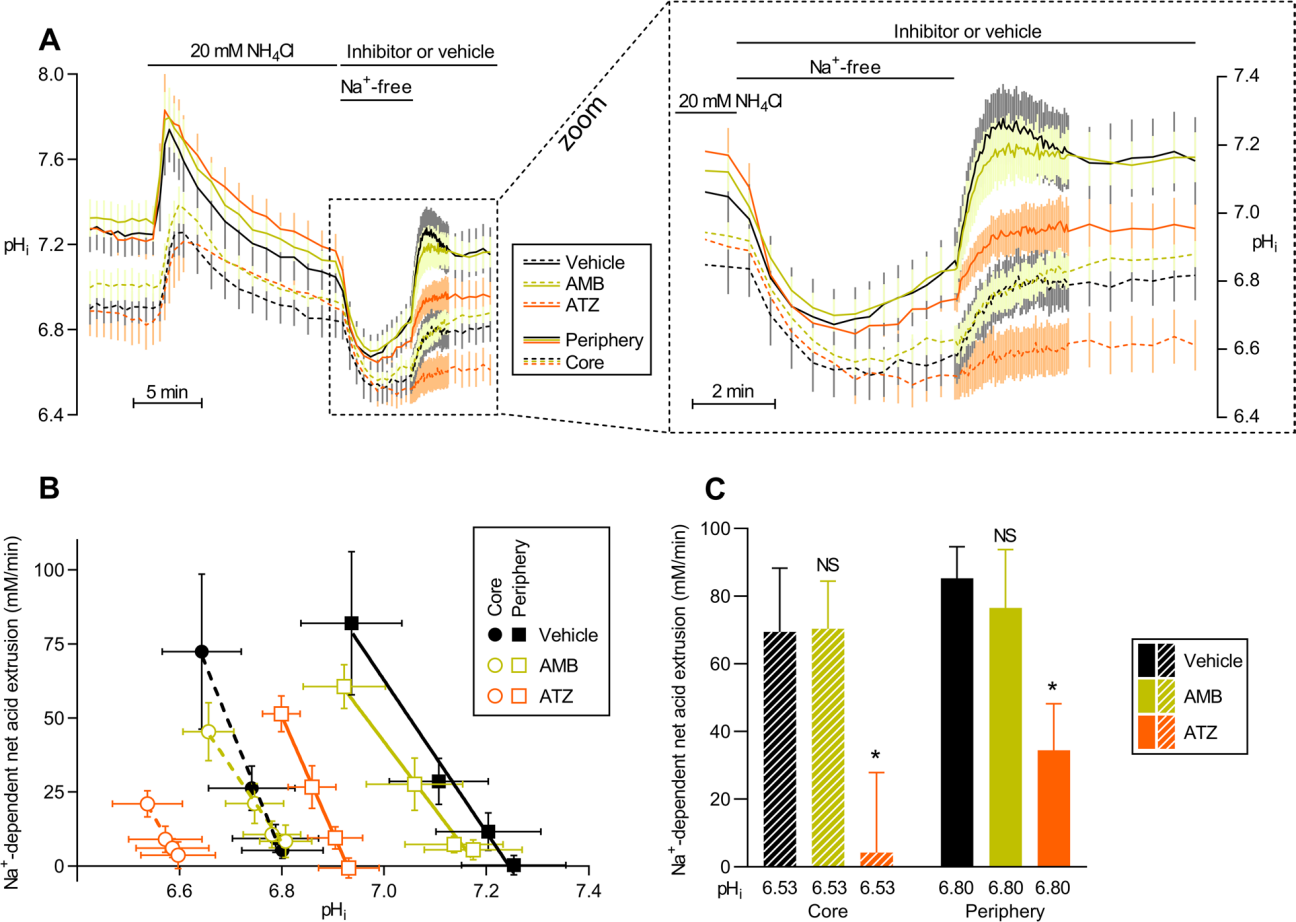
Inhibition of intracellular carbonic anhydrases lowers the capacity for cellular net acid extrusion in murine ErbB2-induced breast cancer tissue. **A** Average curves of pH_i_ in freshly isolated breast cancer organoids during NH_4_^+^-prepulse-induced intracellular acidification and subsequent recovery (n = 6). Experiments were performed with acetazolamide (ATZ, 100 µM), 4-(aminoethyl)benzenesulfonamide (AMB, 30 µM), or equivalent volume of DMSO vehicle. The right part of the panel shows the recovery phase from intracellular acidification magnified. **B** Net acid extrusion plotted as function of pH_i_ for peripheral and core regions of organoids (cf. Figure 6C) freshly isolated from ErbB2-induced murine breast cancer tissue (n = 6). **C** Net acid extrusion calculated at a fixed pH_i_ corresponding to the initial pH_i_ recovery phase during maximal acidification (n = 6). The values refer to the phase after Na^+^ was added to the bath to activate Na^+^-dependent transport mechanisms. Data were compared by repeated-measures two-way ANOVA followed by Dunnett’s post-tests. *P < 0.05, NS: not significantly different *vs.* vehicle

Whereas acetazolamide and AMB have approximately similar effects on pH_o_ (Fig. 6D-H), their influence on pH_i_ is very different (Fig. 6I-N), with acetazolamide having the more pronounced effects. To evaluate further the influence of intra- and extracellular carbonic anhydrases on the cellular net acid extrusion capacity, we next study the recovery of pH_i_ from intracellular acidification induced by NH_4_^+^-prepulses [29]. As illustrated in Fig. 7A, addition of NH_4_Cl to the solution bathing the organoids creates a transient intracellular alkalinization, when NH_3_ enters cells, followed by gradual recovery, as NH_4_^+^ enters cells more slowly and net base extrusion is activated. When we finally remove NH_4_Cl from the bath, NH_3_ quickly escapes the cells, leaving the imported H^+^ behind and causing the desired intracellular acidification (Fig. 7A). We observe that acetazolamide very prominently inhibits the rate of pH_i_ recovery (Fig. 7A) and corresponding net acid extrusion (Fig. 7B, C) in response to NH_4_^+^-prepulse-induced intracellular acidification, particularly at the organoid core. In contrast, AMB has no significant influence on net acid extrusion under these conditions (Fig. 7A-C).

### Carbonic anhydrases limit acidification of the tumor microenvironment

**Fig. 8:**
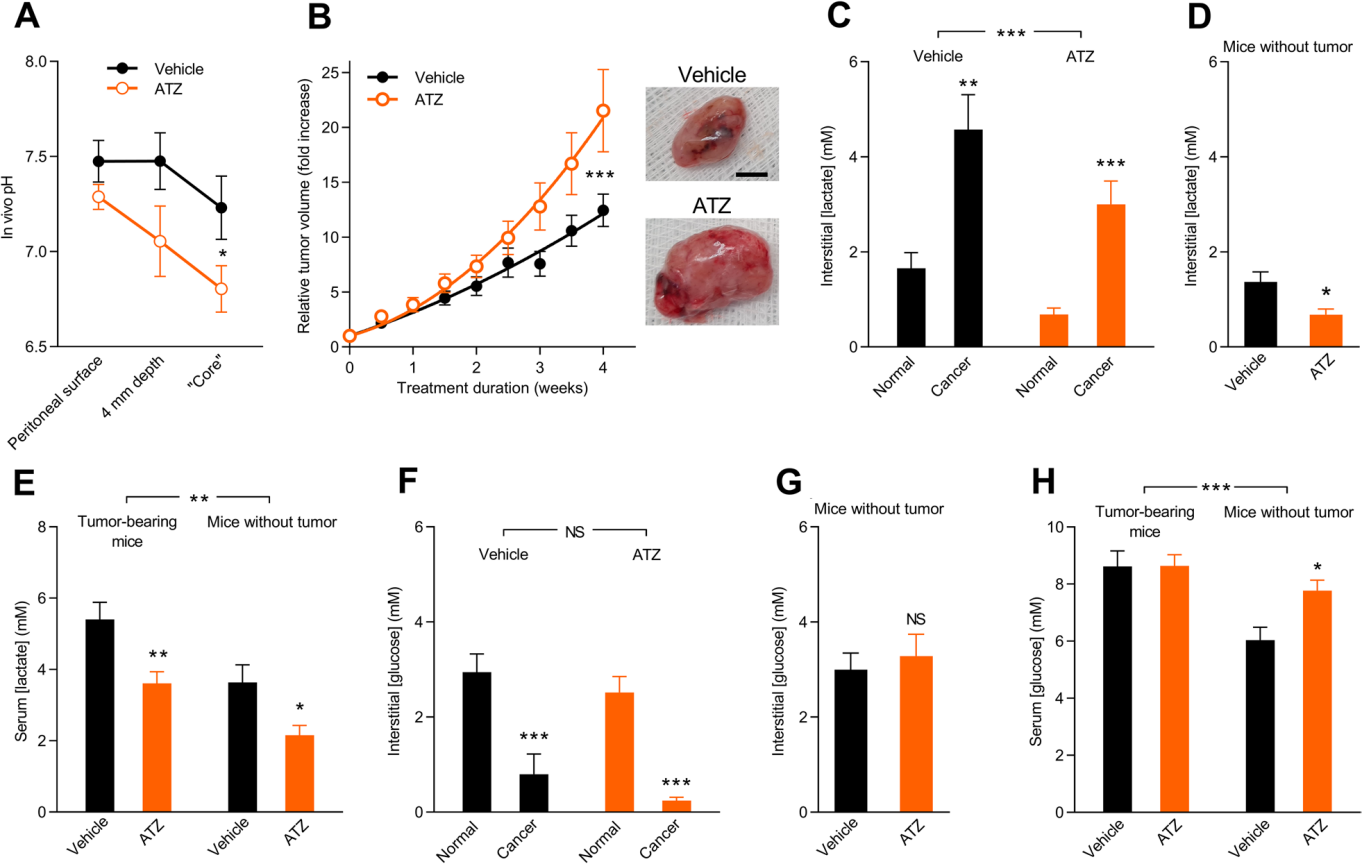
In vivo treatment with acetazolamide acidifies the microenvironment of murine ErbB2-induced breast cancer tissue, lowers lactate concentrations, and accelerates tumor growth. **A** In vivo pH measurements performed with a pH-sensitive microelectrode (n = 8). “Core” was defined as the most acidic region encountered during stepwise impalement with the electrode. The mice had been treated with an intraperitoneal injection of 50 mg/kg acetazolamide (ATZ) or equivalent volume of vehicle 30 min prior to recordings. Data were compared by repeated-measures two-way ANOVA. **B** Exemplary *postmortem* tumor images and in vivo ErbB2-induced breast cancer growth curves for mice treated with 40 mg/kg ATZ by daily intraperitoneal injections compared to mice receiving equivalent volume of vehicle (n = 17). Data were fitted to a second order polynomial function, and the best-fit parameters were compared by extra sum-of-squares *F*-test. The scale bar represents 5 mm; both images are shown at the same magnification. **C–H** Tumor glycolytic metabolism evaluated by microdialysis. Interstitial or serum [lactate] (**C**–**E**) and [glucose] (**F**–**H**) measured 1 h after initiation of ATZ or vehicle administration (n = 8–13). Data were compared by two-way ANOVA followed by Šidák’s post-test (panel **C**,** E**,** F**, and** H**) or unpaired two-tailed Student’s *t* tests (panel **D** and** G**). *P < 0.05, **P < 0.01, ***P < 0.001, NS: not significantly different *vs.* normal tissue (panel **C** and** F**), vehicle (panel **A**,** B**,** D**,** E**,** G**, and** H**), or as indicated

To test whether acetazolamide treatment in vivo indeed leads to acid accumulation in the tumor microenvironment, we next measure pH by progressively moving a pH-sensitive electrode from the peritoneal surface deeper and deeper into the tumor. As shown in Fig. 8A, the degree of tumor acidification is substantially greater in mice treated with acetazolamide (minimum pH 6.8) compared to vehicle (minimum pH 7.2).

### Carbonic anhydrases limit ErbB2-induced breast cancer growth

Inhibition of net acid extrusion can reduce the rate of breast cancer tumor growth [7, 8, 55]; yet, because of the accompanying decrease in pH_o_ in response to carbonic anhydrase inhibition, the net consequences of acetazolamide on breast carcinomas are difficult to predict.

When we treat mice with daily injections of acetazolamide, we observe that the breast tumors grow at a significantly faster rate than tumors in vehicle-treated mice (Fig. 8B). During the 4-week treatment period, tumors in acetazolamide-treated mice undergo a more than 20-fold increase in volume, whereas tumors in vehicle-treated mice increase their volume only around tenfold (Fig. 8B).

### Carbonic anhydrases raise glycolytic metabolism in vivo

The pH values we record from the tumor microenvironment of untreated ErbB2-induced breast carcinomas (Fig. 8A) are high relative to our previous measurements of approximately 6.7 from carcinogen-induced tumors [36]. We previously documented a correspondingly smaller [lactate]-to-[glucose] ratio in ErbB2-induced breast carcinomas relative to carcinogen-induced breast carcinomas [7, 8], which is indicative of lower fermentative glycolysis and can explain the difference in tumor acidification.

In order to evaluate further the composition of the tumor microenvironment and how it is influenced by acetazolamide in mice carrying ErbB2-induced breast carcinomas, we collect blood samples and microdialysates from probes inserted into tumors and matched normal breast tissue. As expected, lactate concentrations are higher (Fig. 8C) and glucose concentrations lower (Fig. 8F) in the tumor tissue than in normal breast tissue. Importantly, acetazolamide treatment lowers [lactate] in the breast cancer tissue, normal breast tissue and in serum from tumor-bearing mice as well as control mice without tumors (Fig. 8C-E). Acetazolamide has no consistent effect on glucose levels across the investigated tissue and blood samples (Fig. 8F-H) except for an increase in serum glucose concentration in normal control mice (Fig. 8H). The similar pattern of effects in breast cancer tissue, normal breast tissue, and serum and between tumor-bearing mice and mice without breast tumors (Fig. 8C-H) suggests that the actions of acetazolamide on metabolism extend beyond cancer tissue.

Considering the lower concentrations of lactate measured in the tumor microenvironment of acetazolamide-treated mice, the increased tumor acidity is not explained by enhanced metabolic acid production but rather by inhibited facilitated diffusion of acid equivalents on the CO_2_/HCO_3_^–^ buffer. On the other hand, the more pronounced acidity—locally in tumors (Fig. 8A) and systemically due to CO_2_ accumulation and urinary loss of HCO_3_^–^—can inhibit glycolytic activity [56] and hence lower lactate levels across tissues.

### Carbonic anhydrases do not influence ErbB2-induced cell proliferation

**Fig. 9:**
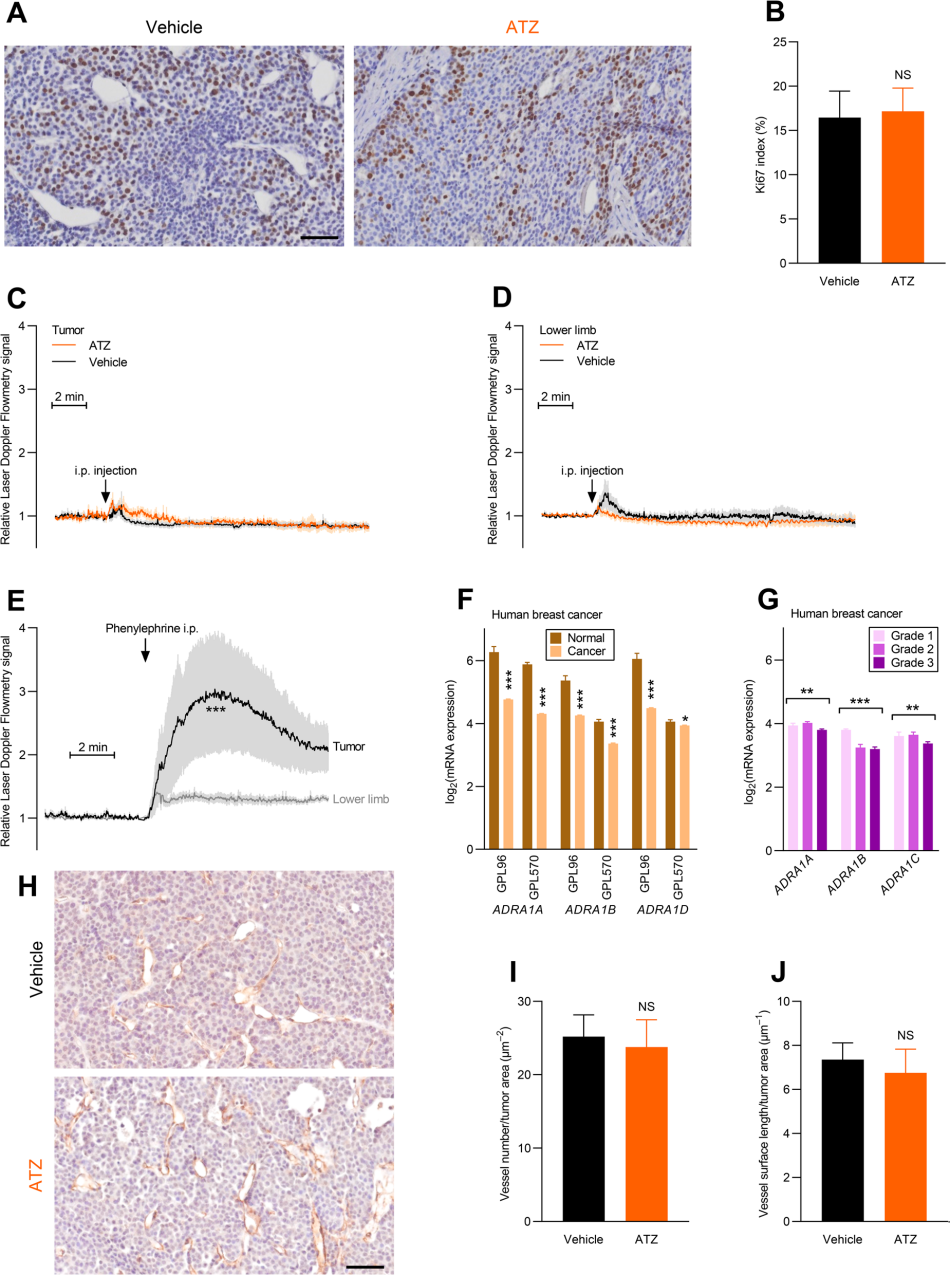
Acetazolamide does not substantially influence cancer cell proliferative activity, tumor blood flow or tumor vascularization. **A + B** Exemplary images (**A**) and quantified Ki67 index (**B**, n = 14–15) in slices of breast carcinomas from mice treated with acetazolamide (ATZ) or vehicle. Scale bar represents 50 µm, and both images are at the same magnification. **C + D** Blood flow responses in breast cancer tissue (**C**) and the vasculature to the lower limb (**D**) during intraperitoneal injection of acetazolamide or vehicle (n = 5–6). **E **Blood flow responses in breast cancer tissue and the vasculature to the lower limb during intraperitoneal injection of the α_1_-adrenoceptor agonist phenylephrine (n = 5). **F + G** Expression of α_1_-adrenoceptors in breast cancer tissue compared to normal breast tissue (**F**, n = 92–5574) and in breast cancer tissue of increasing malignancy grade (**G**, n = 82–446). Data from the GPL96 and GPL570 platforms were extracted from the GENT2 database [38]. **H-J** Exemplary images and quantification of CD105-positive tumor vessels in slices of breast cancer tissue (n = 9–10). Scale bar represents 50 µm, both images are at the same magnification. Data in panel **B**,** F**,** I**, and** J** were compared by unpaired two-tailed Student’s *t* tests. Data in panel **E** were compared by repeated-measures two-way ANOVA. Data in panel **G** were compared by one-way ANOVA for trend followed by Holm–Bonferroni adjustment for multiple comparisons (see Additional file 1: Table S5 for details). *P < 0.05, **P < 0.01, ***P < 0.001, NS: not significantly different *vs.* vehicle (panel **B**,** I**, and** J**), lower limb (**E**), normal tissue (panel **F**), or across malignancy grades as indicated (panel **G**)

Inhibition of net acid extrusion and decreases in pH_i_ typically decelerate cell proliferation in breast carcinomas [7, 8], and the pH_i_ consequences of acetazolamide are therefore unlikely to favor tumor growth. Nonetheless, we next explore whether the increased tumor growth rate in response to acetazolamide is accompanied by a change in proliferative activity. We evaluate cell proliferation based on immunohistochemical staining for the mitotic marker Ki67 and find no overall difference in the proportion of Ki67-positive cells between tumors treated with acetazolamide and vehicle (Fig. 9A, B). The heterogeneous pattern of cell proliferation likely contributes to this observation because even in vehicle-treated mice the large majority of cell divisions take place in regions near blood vessels (Fig. 9A) where acetazolamide—based on our ex vivo organoid experiments (Figs. 6 and 7)—is expected to less prominently affect pH compared to deeper and more poorly perfused tumor regions.

### Carbonic anhydrase inhibitors do not acutely alter tumor perfusion or influence vascularization

Tumor perfusion plays a key role in providing O_2_ and substrate for metabolism and eliminating metabolic waste products. Acetazolamide can induce transient vasocontraction during increases in pCO_2_ and more sustained vasorelaxation, with some variability between vascular beds, but the mechanisms of action and dependency on pH are disputed [33, 57, 58]. We perform Laser Doppler Flowmetry to evaluate tissue perfusion but observe no convincing influence of acetazolamide on perfusion of the breast cancer tissue or the hind limb (Fig. 9C, D).

We have previously demonstrated that tumor feed arteries supplying ErbB2-induced breast carcinomas are specialized toward heightened perfusion as they have lower α_1_-adrenoceptor expression and show lower responsiveness to noradrenaline and electrical field stimulation of sympathetic nerve endings [22]. Consistent with this functional specialization—and supporting that meaningful changes in tumor perfusion are easily detectable by the applied Doppler Flowmetry technique—we show that injection of the α_1_-adrenoceptor agonist phenylephrine causes a higher increase in perfusion of the tumor compared to the hind limb (Fig. 9E). The overall increase in perfusion of both the tumor and peripheral tissue in response to a vasoconstrictor agent suggests an elevation of cardiac output that can be explained by elevated preload due to phenylephrine-induced venoconstriction [59, 60]. Based on human transcriptomic data, we also confirm that the reduced α_1_-adrenoceptor expression previously reported for mice [22] is also observed in human breast cancer tissue, especially of higher malignancy grade (Fig. 9F, G and Additional file 1: Table S5).

The tumor vasculature responds to changes in the tumor microenvironment [61]. Based on immunohistochemical staining for the endothelial cell marker CD105, we therefore evaluate whether carbonic anhydrase inhibition influences the number and sizes of tumor blood vessels (Fig. 9H-J). Relative to vehicle, treatment with acetazolamide for 4 weeks does not influence the density (Fig. 9I) or size (Fig. 9J) of the tumor blood vessels.

### Carbonic anhydrases promote tumor immune infiltration and cytokine expression in ErbB2-induced breast carcinomas

**Fig. 10:**
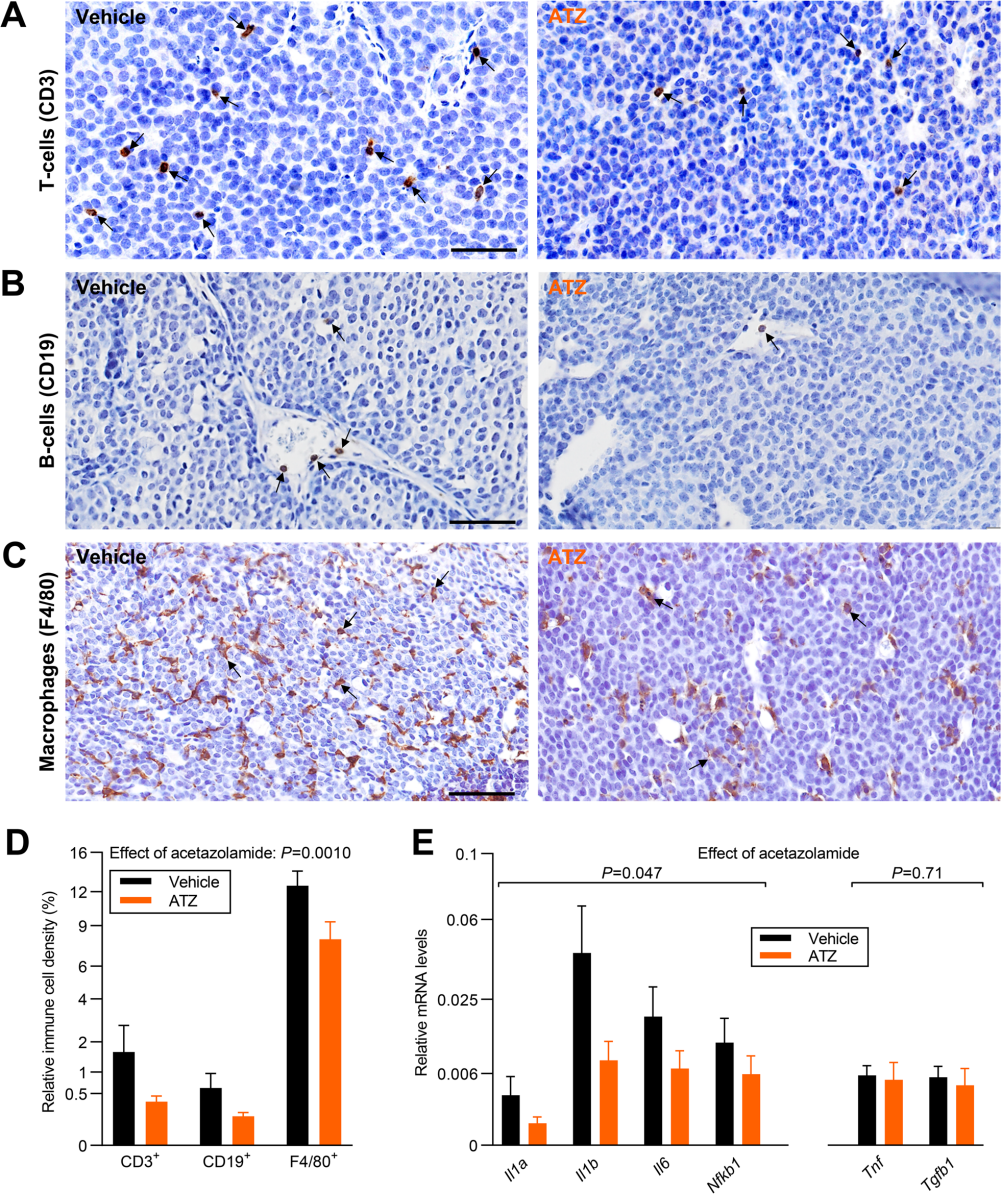
Acetazolamide lowers tumor immune cell infiltration and inflammation. **A-C** Exemplary immunohistochemical images of tumor sections stained for CD3 (T cells, **A**), CD19 (B cells, **B**), and F4/80 (macrophages, **C**). Tumors were from mice treated with acetazolamide (ATZ) or vehicle. Scale bars are 50 µm, all images are at same magnification. Arrows indicate positive cells; in panel **C**, only few are highlighted due to the high number of positive cells. **D** Quantification of the relative density of immune cells within tumors (n = 12–15). We quantified cells within the epithelial tumor mass and intraepithelial connective tissue strands. **E** Expression data for inflammatory cytokines and transcription factors. Following square root transformation, data were compared by repeated-measures two-way ANOVA and the overall effects of ATZ are reported

The chemical composition of the tumor microenvironment has potential to modify anti-tumor immune responses [1]. In order to evaluate immune infiltration, we immunohistochemically stain sections of the murine ErbB2-induced breast carcinomas for CD3^+^ T cells, CD19^+^ B cells, and F4/80^+^ macrophages (Fig. 10A-C). The density of macrophages is high, T cells moderate, and B cells low; yet overall the abundance of these cell types is reduced by around half in breast cancer tissue from mice treated with acetazolamide compared to vehicle (Fig. 10D).

We next test cytokine expression in the breast cancer tissue (Fig. 10E). Previous studies show that HER2 overexpression triggers a pro-inflammatory response involving IL1, IL6, and downstream signaling mechanisms including NFκB [62]. Indeed, we find that vehicle-treated ErbB2-induced breast cancer tissue expresses *Il1a*, *Il1b*, *Il6*, and *Nfkb1* at a measurable level (Fig. 10E). Furthermore, in tumors from mice treated with acetazolamide, the expression level for these pro-inflammatory markers is reduced (Fig. 10E). In contrast, we find that *Tnfa* and *Tgfb1* expression is unaffected by acetazolamide treatment (Fig. 10E).

**Fig. 11:**
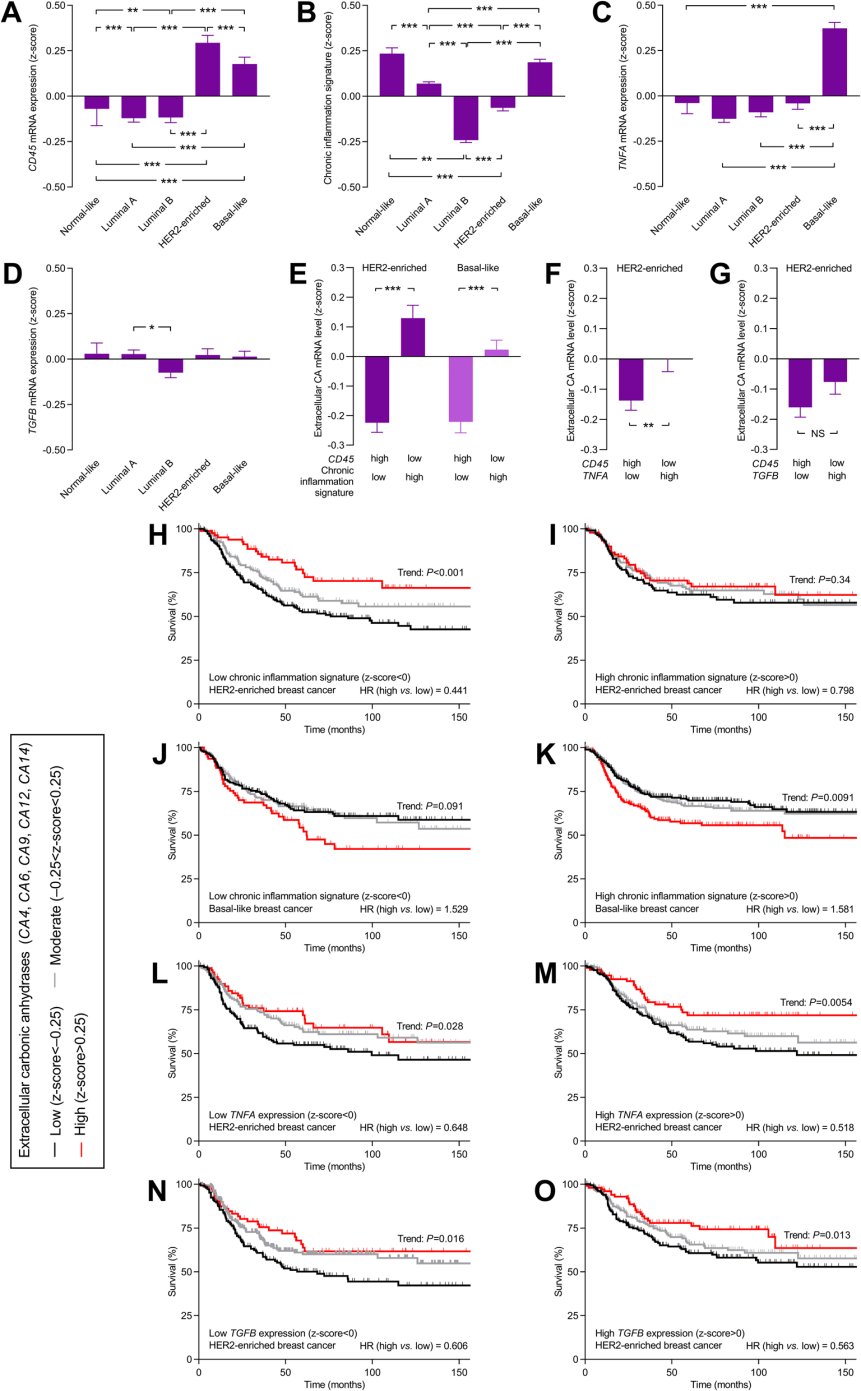
The chronic inflammatory profile of human breast cancer tissue reflects the expression of extracellular carbonic anhydrases and influences their relation to patient survival in HER2-enriched breast cancer. **A-D** Expression levels for markers of leucocytes (*CD45*; **A**) and chronic inflammation (*IL1A*, *IL1B*, *IL4*, *IL6*, *NFKB1*, *STAT3*, *CXCL2*; **B**) and the levels of tumor necrosis factor α (*TNFA*; **C**) and transforming growth factor β (*TGFB*; **D**) across breast cancer molecular subtypes (n = 108–2053). **E–G** Expression of extracellular carbonic anhydrases in breast carcinomas characterized either by high immune infiltration (*CD45* expression) and low inflammation or by low immune infiltration and high inflammation (n = 98–235). **H–O** Survival curves for patients with HER2-enriched (**H**, **I**, **L**-**O**) or Basal-like (**J**, **K**) breast cancer (n = 382–738) stratified for inflammatory markers and expression of extracellular carbonic anhydrases. The ticks on the curves indicate censored subjects. The chronic inflammation signature was calculated as the average z-score for *IL1A*, *IL1B*, *IL4*, *IL6*, *NFKB1*, *STAT3*, and *CXCL2*. Data were compared by one-way ANOVA followed by Šidák’s post-tests (panels **A–D**), unpaired two-tailed Student’s *t* tests (panels **E–G**) or log-rank tests (panel **H–O**). CA, carbonic anhydrase

To explore further the role of the immune system and the possible interactions between chronic inflammation and carbonic anhydrase-dependent changes in the microenvironment, we return to the human transcriptomic datasets (Fig. 11). Initially, we observe that *CD45* expression—which correlates with leucocyte infiltration [63]—among the breast cancer molecular subtypes is highest in HER2-enriched breast cancer (Fig. 11A). Based on the IL1 pro-inflammatory circuit proposed to drive HER2-dependent tumorigenesis [62], we then define a chronic inflammation signature calculated as the average expression level of *IL1A*, *IL1B*, *IL4*, *IL6*, *CXCL2, NFKB1*, and *STAT3*. Interestingly, despite the high *CD45* expression in HER2-enriched breast cancer (Fig. 11A), the chronic inflammation signature (Fig. 11B) as well as the expression of *TNFA* (Fig. 11C) and *TGFB* (Fig. 11D) is low or average in HER2-enriched breast cancer relative to the other molecular subtypes.

We speculate that the discrepancy between tumor immune infiltration and cytokine expression may relate to the composition of the tumor microenvironment. To test this within HER2-enriched and Basal-like breast cancers, we identify two extreme groups of tumors: one with high expression of *CD45* (indicating intense immune infiltration) but low chronic inflammation markers and an opposite group with low expression of *CD45* (indicating low immune infiltration) but high chronic inflammation markers. Supporting that carbonic anhydrases have pro-inflammatory actions that promote immune cell function—most likely by limiting acidification of the tumor microenvironment—we observe that the expression level of extracellular carbonic anhydrases is higher in the groups with high chronic inflammation but low immune infiltration compared to the groups with low chronic inflammation but high immune infiltration (Fig. 11E). The difference in extracellular carbonic anhydrase expression is 3 times bigger when we divide tumors based on the chronic inflammation signature (Fig. 11E) rather than *TNFA* (Fig. 11F), and we observe no significant difference in extracellular carbonic anhydrase expression when we divide the tumors based on the level of *TGFB* expression (Fig. 11G).

### Effects of carbonic anhydrase expression on patient survival are immune status-dependent

The expression of extracellular carbonic anhydrases has stronger predictive power for survival of patients suffering from HER2-enriched breast cancer with low (HR = 0.441; Fig. 11H) than high (HR = 0.798; Fig. 11I) chronic inflammation signature. In contrast, we observe no substantial influence of the chronic inflammation signature on the predictive power of extracellular carbonic anhydrases in Basal-like breast cancer (HR = 1.581 and 1.529; Fig. 11J, K) underscoring the different consequences of carbonic anhydrases in these two breast cancer molecular subtypes.

Notably, we also see no substantial influence of the mRNA levels of *TNFA* (HR = 0.648 *vs.* 0.518) or *TGFB* (HR = 0.606 *vs.* 0.563) on the survival effects of carbonic anhydrases (Fig. 11L-O) in HER2-enriched breast cancer.

Together these findings reveal that survival among patients with HER2-enriched breast cancer is very sensitive to the degree and nature of the inflammatory response. Overall, survival is shorter in patients with breast carcinomas of low chronic inflammation (compare Fig. 11H, I), particularly when combined with low carbonic anhydrase expression (Fig. 11H). In contrast, our data support that the negative influence of reduced pro-inflammatory signaling can be overcome when extracellular carbonic anhydrase expression is high (Fig. 11H) and, predictably, the tumor microenvironment is less acidic.

## Discussion

Acid–base reactions—typified by the exchange of H^+^ ions—are fast and do not require enzymatic catalysis. Yet, the spontaneous interconversion between CO_2_ and HCO_3_^–^ through the reactions CO_2_ + H_2_O $\rightleftarrows$ H_2_CO_3_ $\rightleftarrows$ HCO_3_^–^ + H^+^ relies on slow reversible hydration of CO_2_ to carbonic acid. Carbonic anhydrases circumvent the issue of slow CO_2_ hydration as they catalyze the reaction CO_2_ + H_2_O $\rightleftarrows$ HCO_3_^–^ + H^+^ [64] permitting the CO_2_/HCO_3_^–^ buffer to powerfully minimize local pH deviations in response to acid or base loads and increase the effective CO_2_ and H^+^ mobility.

In the current study, we comprehensively explore the contribution of carbonic anhydrases in breast cancer tissue. Based on acute studies on human and murine breast cancer biopsies, we show that (a) intracellular carbonic anhydrases accelerate cellular net acid extrusion, whereas (b) extracellular carbonic anhydrases facilitate H^+^ elimination in the interstitial space from deeper diffusion-restricted tumor regions to the tumor periphery. In an immunocompetent syngeneic mouse model of ErbB2-induced breast cancer, we next show that carbonic anhydrase inhibitors (c) acidify the tumor microenvironment, (d) lower immune infiltration and chronic tumor inflammation, and (e) accelerate tumor growth. Finally, we integrate this information with clinically annotated human transcriptomic data supporting that (f) extracellular carbonic anhydrases improve survival of patients with HER2-enriched breast cancer in a manner that depends on the chronic inflammatory profile of the tumor tissue.

Elimination of acidic waste products from solid cancer tissue is a composite process occurring across the organellar, cytosolic, and interstitial compartments (Fig. 1A). To address the roles of carbonic anhydrases in each of these compartments, we group them according to their known subcellular expression patterns (Figs. 3 and 11) and use pharmacological tools that distinguish between intra- and extracellular carbonic anhydrases (Figs. 6 and 7). We find that intracellular carbonic anhydrase activity is critical for net acid extrusion from cancer cells (Fig. 7) and for their ability to elevate pH_i_ (Fig. 6I-N), particularly in tumor regions challenged by diffusion hindrances. This observation is compatible with previous mathematical models [53, 54] showing that cytosolic carbonic anhydrase activity can enhance HCO_3_^–^ uptake across the plasma membrane. According to the models, the importance of the carbonic anhydrases for transmembrane net H^+^ fluxes disappears if the concentration of non-carbonic intracellular mobile buffers and their accessibility to the transporters in the membrane are sufficiently high [53, 54]. Our observations and the results of the mathematical models are in agreement with experimental observations from cardiomyocytes where cytosolic but not extracellular carbonic anhydrase activity facilitates Na^+^,HCO_3_^–^-cotransport activity [54]. In our studies, the acetazolamide-induced inhibition of net acid extrusion (Fig. 7B) is of similar magnitude to that achieved when Na^+^,HCO_3_^–^-cotransport is abolished by knockout of NBCn1 [7, 8].

A previous report suggests that intercellular transfer of acid through gap junctions can also contribute to elimination of cytosolic acid loads [65]. It is conceivable that inhibition of carbonic anhydrase-dependent facilitated transfer of CO_2_ and H^+^ from cell to cell through gap junctions is partly responsible for the amplified core-to-periphery pH_i_ gradient of breast cancer organoids in response to acetazolamide (Fig. 6I-N).

The acid extruded from cancer cells must next be transferred to the bloodstream and ultimately metabolized in oxidative cells (e.g., lactate and H^+^) or eliminated via the lungs (CO_2_) or kidneys (non-volatile acid). The extracellular carbonic anhydrases play a particularly critical role facilitating the interstitial transfer of acid to nearby blood vessels (Fig. 1A), which in the ex vivo experimental setting corresponds to the bath solution (Fig. 6D-H). When carbonic anhydrase inhibition lowers the effective interstitial H^+^ mobility, elimination of the steady-state acid load requires a correspondingly larger pH_o_ gradient. Thus, interpatient differences in carbonic anhydrase expression and interventions to inhibit carbonic anhydrase activity will influence the chemical composition of the tumor microenvironment, particularly in deeper and poorly perfused regions.

Cellular metabolism and acid–base levels are mutually interdependent. Oxidative metabolism and fermentative glycolysis are the main sources of acid loading in tumors, but acidification also regulates metabolic activity via pH-sensitive enzymatic reactions. Phosphofructokinase 1 that catalyzes the committing step of glycolysis is one of the most pH-sensitive enzymes described and displays dramatic inhibition if pH drops even 0.1–0.3 below normal levels [56, 66]. In the current study, we observe that acetazolamide therapy lowers lactate levels throughout the body (in tumors, normal breast tissue, and plasma) and this effect likely reflects universal intracellular acidification due to CO_2_ and H^+^ accumulation and, over time, urinary loss of HCO_3_^–^.

Carbonic anhydrase activity lowers pH_o_ if the catalyzed reaction is in direction of net CO_2_ hydration, which is likely in areas dominated by oxidative phosphorylation (Fig. 1A, upper schematic). Conversely—as illustrated in the lower schematic of Fig. 1A—carbonic anhydrase activity elevates pH_o_ when the net reaction is buffering of H^+^ by HCO_3_^–^, for instance, in areas dominated by fermentative glycolysis [67]. In addition, buffer-assisted diffusion requires the rapid reaction rates provided by carbonic anhydrase activity [68], and if CO_2_/HCO_3_^–^ buffer equilibration is blocked, the effective H^+^ mobility falls and tissue pH_o_ declines. In line with these opposing effects of carbonic anhydrases, their net influence on tissue interstitial pH varies [11, 69, 70] although most previous cancer studies evaluate conditions where carbonic anhydrases promote extracellular acidification.

Based on our recordings from breast cancer tissue—evaluated as primary organoids (Fig. 6) and in vivo (Fig. 8A)—carbonic anhydrase activity counteracts both intra- and extracellular acidosis. These observations support the quantitative prominence of extracellular carbonic anhydrases in buffering H^+^ from fermentative glycolysis and facilitating H^+^ and CO_2_ diffusion from the cancer cells to the bloodstream. When the effective H^+^ mobility is reduced and the capacity for net acid extrusion is lowered following carbonic anhydrase inhibition (Fig. 7), a larger pH gradient along the diffusive path and a greater degree of intracellular acidification are required for extrusion of the acid load at steady state (Fig. 6). The intracellular acidification may reduce metabolic acid production (Fig. 8C); but even if the interstitial space receives a smaller acid load, an amplified pH_o_ gradient from the core to the periphery of tumor tissue (Fig. 6F, H) is required for eliminating this acid load due to the lower effective H^+^ mobility. Studying primary organoids ex vivo and tumors in vivo—rather than superfused isolated cells or 2-dimensional cultures—allows us to integrate the extra- and intracellular diffusion limitations with changes in metabolism and transport activities imposed by carbonic anhydrase inhibition.

Blood flow control in tumors differs substantially from normal tissue; as tumor blood vessels are both structurally and functionally specialized toward low vascular resistance [22, 71]. Indeed, we show here that infusion of an α_1_-adrenoceptor agonist—in line with the characteristically low contractile responsiveness of isolated tumor blood vessels [22]—results in a markedly larger blood flow increase in tumors compared to other tissues (Fig. 9E). These same tumor blood vessels, however, show no discernible response to acetazolamide (Fig. 9C, D) supporting that the altered interstitial accumulation of metabolites (Fig. 8C-H) and H^+^ (Fig. 8A) following acetazolamide therapy is not explained by effects on tumor perfusion. The phenylephrine-induced increase in blood flow despite vasoconstriction indicates that concomitant venoconstriction increases preload and cardiac output [59, 60].

Combining intervention studies in mice with analyses of clinically annotated transcriptomic data, we provide evidence that carbonic anhydrases and their pharmacological inhibitors can change the trajectory of cancer progression. Most prominently, we observe that carbonic anhydrase expression and activity can explain differences in tumor immune cell infiltration (Fig. 10A-D) and chronic inflammation (Figs. 10E and 11E). Low expression of extracellular carbonic anhydrases—functionally equivalent to experimental carbonic anhydrase inhibition, which is associated with acidosis in breast cancer tissue (Fig. 6)—characterizes tumors with reduced expression of inflammatory cytokines (*Il1A*, *IL1B*, *IL4*, *IL6*, *CXCL2*) and transcription factors (*NFKB1*, *STAT3*) relative to the degree of tumor immune cell infiltration (Fig. 11E). Conversely, we observe high expression of extracellular carbonic anhydrases in tumors with an elevated chronic inflammatory signature relative to tumor immune cell infiltration (Fig. 11E). These findings are in line with previous reports that acidosis inhibits immune cell function [21].

Carbonic anhydrases strongly predict survival of patients with HER2-enriched breast cancer displaying low (Fig. 11H) but not high (Fig. 11I) levels of pro-inflammatory cytokines and transcription factors in the tumor tissue. The overall survival of patients suffering from HER2-enriched breast cancer with high chronic inflammatory signatures, regardless of the carbonic anhydrase expression level, is very similar to that in less inflamed tumors with high carbonic anhydrase levels (compare Fig. 11H, I). These observations suggest that carbonic anhydrases—likely via their ability to elevate tumor pH_o_ (Figs. 6 and 8A)—provide an immune-stimulatory input that improves survival of patients with HER2-enriched breast cancer characterized by a weak immune response. We propose that an additional pro-inflammatory signal from carbonic anhydrase-dependent alkalinization is critical for the survival of the patients with poorly immunogenic tumors (Fig. 11H), whereas carbonic anhydrases are not necessary for mounting an effective immune response in the more immunogenic tumors (Fig. 11I).

In contrast to HER2-enriched breast cancer, the association between extracellular carbonic anhydrase expression and patient survival in Basal-like breast cancer is seemingly unaffected by tumor inflammation (Fig. 11J, K). These observations can, at least in part, explain the very different associations between carbonic anhydrase expression and breast cancer progression and prognosis in HER2-enriched and Basal-like breast cancer (Fig. 3). The findings also underscore the importance of performing experimental cancer studies in immunocompetent models integrating direct effects on cancer cells with indirect effects via the stromal tumor component.

Considering the faster growth of ErbB2-induced breast carcinomas in response to carbonic anhydrase inhibition (Fig. 8B) and the positive predictive value of carbonic anhydrases in HER2-enriched breast cancer (Fig. 3C, G), the current study does not support use of carbonic anhydrase inhibitors for treatment of HER2-enriched breast cancer. On the contrary, our study highlights that caution is called for in patients receiving long-term therapy with carbonic anhydrase inhibitors for other indications. Acetazolamide was classically used as a diuretic and is in current clinical use for glaucoma, idiopathic intracranial hypertension, seizures, congestive heart failure, and mountain sickness [10–12]. Whereas it is likely that patients with Basal-like breast cancer could benefit from CA9- and CA13-targeted therapy (Fig. 5), our current findings show that patients with HER2-enriched breast cancer will likely follow a more severe disease trajectory if treated with a carbonic anhydrase inhibitor. This is important information considering the wide clinical indications for acetazolamide and the high incidence of breast cancer, which makes it likely that these patient groups overlap. The different predictive values of carbonic anhydrases in HER2-enriched and Basal-like breast cancer exemplify the heterogeneity of breast cancer and the need for personalized treatment decisions to improve therapeutic responses and lower the risk of harm.

The overall survival benefit associated with high expression of extracellular carbonic anhydrases in patients with HER2-enriched breast cancer (HR = 0.581; Fig. 3C) is well within the clinically relevant range. Strikingly, in fact, the effect size is similar to the therapeutic benefit of the anti-HER2 antibody trastuzumab (HR = 0.64 [72]) in a similar patient population. Whereas our current approach focuses across the breadth of the 12–13 catalytically active members of the carbonic anhydrase family, future studies should explore the role of individual carbonic anhydrases that could be targeted therapeutically. Ongoing medicinal chemistry endeavors attempt to develop new compounds that can inhibit or activate specific carbonic anhydrases without systemic adverse effects [12, 13, 73].

## Conclusions

We show that carbonic anhydrases (a) elevate pH in breast carcinomas by accelerating net H^+^ elimination from cancer cells and across the interstitial space, (b) raise immune infiltration and inflammation and decelerate growth of ErbB2-induced breast carcinomas, and (c) improve survival specifically for patients with HER2-enriched breast cancer.

## Supplementary Information

**Additional file 1.** Supplementary tables and figures.** Table S1** summarizes the clinical and pathological patient characteristics.** Table S2 and S3** provide sequence information for primers and probes used for quantitative RT-PCR analyses.** Table S4 and S5** give detailed information on the statistics analyses in Figs. 1 and 9.** Figure S1** shows patterns of gene expression for carbonic anhydrases across breast cancer molecular subtypes.** Figure S2 and S3** provide survival curves for Luminal A (Fig. S2) and Luminal B (Fig. S3) breast cancer patients stratified for carbonic anhydrase expression.

## Data Availability

The human proteomic and transcriptomic data with associated clinicopathologic information are available from public repositories or previous publications as specified in the Materials and Methods section. The experimental data from mice and humans are available in de-identified form from the corresponding author upon reasonable request.

## Abbreviations

AMB: 4-(Aminomethyl)benzenesulfonamide
ATZ: Acetazolamide
BCECF: 2′,7′-Bis-(2-carboxyethyl)-5-(and-6)-carboxyfluorescein
CA: Carbonic anhydrase
DAB: 3,3′-Diaminobenzidine
HER2: Human epidermal growth factor receptor 2
HR: Hazard ratio
L: Length
MCT: Monocarboxylate transporter
PBS: Phosphate-buffered saline
pH_i_: Intracellular pH
pH_o_: Extracellular pH
V: Volume
W: Width
