# Supplementary material for: Carbonic anhydrases reduce the acidity of the tumor microenvironment, promote immune infiltration, decelerate tumor growth, and improve survival in ErbB2/HER2-enriched breast cancer
Source: Breast Cancer Res. 2023 Apr 25;25:46. doi: 10.1186/s13058-023-01644-1 (PMC10127511; doi:10.1186/s13058-023-01644-1)
Supplement: Supplementary file 1 — Additional file 1. Supplementary tables and figures. Table S1 summarizes the clinical and pathological patient characteristics. Table S2 and S3 provide sequence information for primers and probes used for quantitative RT-PCR analyses. Table S4 and S5 give detailed information on the statistics analyses in Figs. 1 and 9. Figure S1 shows patterns of gene expression for carbonic anhydrases across breast cancer molecular subtypes. Figure S2 and S3 provide survival curves for Luminal A (Fig. S2) and Luminal B (Fig. S3) breast cancer patients stratified for carbonic anhydrase expression. [file 13058_2023_1644_MOESM1_ESM.docx]

**Supplementary Material for**

**Carbonic anhydrases reduce the acidity of the tumor microenvironment, promote immune infiltration, decelerate tumor growth, and improve survival in ErbB2/HER2-enriched breast cancer**

Soojung Lee^1,*^, Nicolai J. Toft^1,*^, Trine V. Axelsen^1^, Maria Sofia Espejo^1^, Tina M. Pedersen^1^, Marco Mele^2^, Helene L. Pedersen^3^, Eva Balling^2^, Tonje Johansen^3^, Mark Burton^4,5,6^,
Mads Thomassen^4,5^, Pernille Vahl^7^, Peer Christiansen^2,8^, Ebbe Boedtkjer^1^

^1^Department of Biomedicine, Aarhus University, Aarhus, Denmark, ^2^Department of Surgery, Randers Regional Hospital, Randers, Denmark, ^3^Department of Pathology, Randers Regional Hospital, Randers, Denmark, ^4^Department of Clinical Genetics, University of Southern Denmark, Odense, Denmark, ^5^Clinical Genome Center, University and Region of Southern Denmark, Odense, Denmark, ^6^Department of Clinical Medicine, University of Southern Denmark, Odense, Denmark, ^7^Department of Pathology, Aarhus University Hospital, Aarhus, Denmark, ^8^Department of Plastic and Breast Surgery, Aarhus University Hospital, Aarhus, Denmark.

*these authors share the first authorship

**Supplementary Tables**

| Number of patients | 23 |
| --- | --- |
| Patient age (years; median, interquartile range) | 68.5 (62.3-74.8) |
| Tumor size (mm; median, interquartile range) | 15.5 (13.3-20.8) |
| Histological type  Invasive ductal carcinoma  Invasive lobular carcinoma  Combined invasive ductal and lobular carcinoma | 86%  9%  5% |
| HER2 receptor status  Normal  Overexpression or gene amplification | 95%  5% |
| Estrogen receptor status  Positive  Negative | 95%  5% |
| Malignancy grade  I  II  III | 23%  50%  27% |
| Axillary lymph node status  Negative  Metastasis or isolated tumor cells | 67%  33% |
| Ki67 index  Low (≤15%) positive cells  Moderate (16-30%) positive cells  High (>30%) positive cells | 27%  36%  36% |

**Supplementary Table 1.** Clinical and pathological characteristics of the patients investigated by quantitative RT-PCR and evaluated for pH_i_ and pH_o_ dynamics.

| **Isoform** | **Forward primer (5’ → 3’)** | **Reverse primer (5’ → 3’)** | **Product size (bp)** |
| --- | --- | --- | --- |
| *Car1* | TTGATGACAGTAGCAACC | CCAGTGAACTAAGTGAAG | 161 |
| *Car2* | CAAGCACAACGGACCAGA | ATGAGCAGAGGCTGTAGG | 123 |
| *Car3* | GCTCTGCTAAGACCATCC | ATTGGCGAAGTCGGTAGG | 117 |
| *Car4* | CTCCTTCTTGCTCTGCTG | GACTGCTGATTCTCCTTA | 146 |
| *Car5a* | ACCAAAGCAAGGGCATACAG | TGGCACAGAGAAGTCCCACA | 116 |
| *Car5b* | AATGGCTTGGCTGTGATAGG | GGCGTAGTGAGAGACCCAGA | 188 |
| *Car6* | AAGATTGACGAGTATGCC | TAGGTGTAATAGTGGTGG | 146 |
| *Car7* | CAATGACAGTGATGACAGAA | TCCAGTGAACCAGATGTAG | 161 |
| *Car9* | CTGAAGACAGGATGGAGAAG | GCAGAGTGCGGCAGAATG | 222 |
| *Car12* | CCTATGTTGGTCCTGCTG | CATTGTAACCTTGGAACTG | 144 |
| *Car13* | AATACGACTCCTCACTCC | TGCCGCAACCTGTAGTTC | 160 |
| *Car14* | TCATCCAAGCAGGACCACTG | TCCCAGCCGCTTCTTCCTAA | 131 |
| *Car15* | AGCACAGCCTGGATGAGA | CAGACACAATGGCAGAGA | 171 |
| *Rps18* | GGATGTGAAGGATGGGAAGTACA | TCCAGGTCCTCACGCAGCTTGTT | 72 |
| *Actb* | TGACGTTGACATCCGTAAAG | CTGGAAGGTGGACAGTGAGG | 205 |

**Supplementary Table 2.** Primers for quantitative PCR based on cDNA reverse transcribed from total RNA isolated from mouse normal breast tissue and breast cancer tissue.

| **Isoform** | **Forward primer** **(5’ → 3’)** | **Reverse primer (5’ → 3’)** | **Product size (bp)** |
| --- | --- | --- | --- |
| *CA1* | AAATGAGCATGGTTCAGAACATACA | ACTTTGCAGAATTCCAGTGAGCTA | 83 |
| *CA2* | TGGACTGGCCGTTCTAGGTATT | CCAGCACATCAACAACTTTCTGA | 77 |
| *CA3* | CACACCGTGGATGGAGTCAA | GTGTTATACTTCGGGTTCCAGTGA | 68 |
| *CA4* | ATACCAGGCCAAACAGTTGC | TCGATGTCCCCTTCTCTTTC | 77 |
| *CA5A* | CACCTGGATCATCCAGAAGGA | ACAGGAGAGTACGAAATGCAGAGA | 71 |
| *CA5B* | GCCCTGCAGCCTCTATACTTGT | CACGCTCTCCCAGAGTGGAT | 70 |
| *CA6* | GATGCGCCGGATGGTTT | GTAATAAGTGTTTTCAGGGTAATTCTTCA | 72 |
| *CA7* | CAATGACAGCGATGACCGAA | TCCAGTGAACCAGATGCAG | 72 |
| *CA9* | GAAAACAGTGCCTATGAGCAGTTG | TCCTGGGACCTGAGTCTCTGA | 78 |
| *CA12* | ACTGCGGCAGGACTGAGTCT | CACAATACAGATGCCAAGAATGC | 72 |
| *CA13* | TGGAAGCTACAGGTTACGGC | GCCTCAACAAAGCTGGGGTA | 91 |
| *CA14* | TCAGAACACCAGATCAACAGTGAA | CACTCAAGCTGTCATAGGAATCAGA | 85 |
| *RPS18* | GGATGTAAAGGATGGAAAATACA | TCCAGGTCTTCACGGAGCTTGTT | 72 |
| *ACTB* | CTGGAACGGTGAAGGTGACA | AAGGGACTTCCTGTAACAACGCA | 140 |

**Supplementary Table 3.** Primers for quantitative PCR based on cDNA reverse transcribed from total RNA isolated from human normal breast tissue and matched breast cancer tissue.

| **Gene** | **Slope** | **Adjusted *P*-value (*vs.* slope=0)** |
| --- | --- | --- |
| *CA1* | –0.06876±0.04493 | 0.63 |
| *CA2* | 0.05880±0.08943 | 1.00 |
| *CA3* | –0.05178±0.08775 | 1.00 |
| *CA4* | –0.3500±0.05654 | <0.001 |
| *CA5A* | 0.1013±0.07215 | 0.64 |
| *CA5B* | –0.004623±0.03174 | 1.00 |
| *CA6* | 0.2277±0.07558 | 0.019 |
| *CA7* | –0.1462±0.06799 | 0.19 |
| *CA9* | 0.3813±0.08167 | <0.001 |
| *CA12* | –1.434±0.1024 | <0.001 |
| *CA13* | 0.1961±0.05666 | 0.0048 |
| *CA14* | –0.4639±0.07968 | <0.001 |
| *HIF1A* | 0.5433±0.03982 | <0.001 |
| *SLC2A1* | 0.3941±0.04592 | <0.001 |
| *VEGFA* | 0.2912±0.04645 | <0.001 |
| *MMP1* | 1.878±0.1370 | <0.001 |

**Supplementary Table 4.** Slopes illustrating how the expression of carbonic anhydrases and hypoxia-responsive genes associates with breast cancer malignancy grade, as reported in Figure 1D and E. The displayed *P*-values result from one-way ANOVA for trend and were corrected for multiple comparisons by the Holm-Bonferroni method.

| **Gene** | **Slope** | **Adjusted *P*-value (*vs.* slope=0)** |
| --- | --- | --- |
| *ADRA1A* | –0.1126±0.03666 | 0.0044 |
| *ADRA1B* | –0.3007±0.03921 | <0.001 |
| *ADRA1D* | –0.1624±0.05896 | 0.0060 |

**Supplementary Table 5.** Slopes illustrating how the expression of adrenergic receptors associates with breast cancer malignancy grade, as reported in Figure 9G. The displayed *P*-values result from one-way ANOVA for trend and were corrected for multiple comparisons by the Holm-Bonferroni method.

**Supplementary Figures**

**

**

**Supplementary Figure 1.** Patterns of carbonic anhydrase expression across the different breast cancer molecular subtypes. The expression of *CA9* largely follows that of *HIF1A* and other hypoxia-responsive genes (*VEGFA*, *MMP1*, *SLC2A1*). **P*<0.05, ***P*<0.01, ****P*<0.001, as indicated.

**

**

**Supplementary Figure 2.** Survival curves for each of the carbonic anhydrase isoforms in Luminal A breast cancer. The ticks on the curves indicate censored subjects. Low and high mRNA levels refer to z-scores below and above zero, respectively.

**

**

**Supplementary Figure 3.** Survival curves for each of the carbonic anhydrase isoforms in Luminal B breast cancer. The ticks on the curves indicate censored subjects. Low and high mRNA levels refer to z-scores below and above zero, respectively.
